# Isolation and characterization of a newly discovered plant growth-promoting endophytic fungal strain from the genus *Talaromyces*

**DOI:** 10.1038/s41598-024-54687-5

**Published:** 2024-03-12

**Authors:** Amit C. Kharkwal, Hemesh Joshi, Cheshta Shandilya, Surbhi Dabral, Niraj Kumar, Ajit Varma

**Affiliations:** 1 Amity Institute of Microbial Technology, Amity University Noida, Noida, Uttar Pradesh India; 2 Phymatomics Technologies, Ghaziabad, Uttar Pradesh India

**Keywords:** *Talaromyces albobiverticillius* HNB9, Sustainable agriculture, Fungal root-endophyte, Plant growth promotion, Phosphorus solubilization, Zinc solubilization, Plant–microbe interaction, SDG2, Biological techniques, Biotechnology, Microbiology, Plant sciences, Ecology

## Abstract

In the Kandi zone of Punjab, India, root and rhizospheric soil samples were collected from the local vegetation near the Shivalik mountain foothills. Fifteen fungal colonies exhibiting distinct cultural morphology on Potato Dextrose Agar (PDA) plates were selected for plant–microbe interaction studies. Among these, the isolate HNB9 was identified as a nonpathogenic root colonizer. Morphological and molecular analyses confirmed HNB9 as *Talaromyces albobiverticillius*, characterized by the secretion of a red pigment as a secondary metabolite. Plants colonized with *T. albobiverticillius* HNB9 exhibited enhanced growth, manifesting in increased shoot and root length compared to untreated controls. This study unveiled the first evidence that a species from the *Talaromyces* genus, specifically *T. albobiverticillius*, possesses dual capabilities of root colonization and plant growth promotion. Moreover, HNB9 demonstrated the production of plant growth-regulating compounds like Indole Acetic Acid (IAA) and proficient solubilization of crucial nutrients (Phosphorous, Zinc, and Silica) through plate culture methods. This finding represents a significant contribution to the understanding of root-colonizing fungi with plant growth-promoting attributes, challenging the existing knowledge gap within the *Talaromyces* genus.

## Introduction

The study of plant–microbe interactions has gained significant attention in recent decades due to its economic significance^1,2^. The interaction between microbes and plants relies on various factors, including the type of association with the host plant^3^, the microbe's ability to colonize plant roots^4,5^, and its capacity to solubilize essential nutrients from the surrounding environment^6^. Advanced molecular techniques have revealed the presence of a vast number of microbial species associated with plant roots and leaves^7^. While some microbes remain on the plant's surface as epiphytes, most beneficial microbes typically establish themselves as root endophytes, residing inside plant cells^8^.

Mycorrhizae are fungal root endophytes that form a close mutual symbiosis with nearly all terrestrial plants. They play a crucial role in plant growth promotion, providing essential nutrients such as nitrogen and phosphorus, while also bolstering resistance against both environmental and biological stresses^9–12^. To enhance the growth yield of economically important plant species, numerous mycorrhizal species and mycorrhiza-like fungi are commercially employed. However, a significant obstacle lies in the mycorrhiza's inability to thrive in synthetic mediums^9,13^. Nevertheless, root endophytes like *Serendipita indica* (formerly known as *Piriformospora indica*) possess a remarkable ability to grow in chemically defined mediums and subsequently colonize plants, resulting in improved growth and development^14–16^.

Variable biotopes harbor diverse microorganisms that interact with plants, potentially giving rise to unique and novel secondary metabolites. Extensive research has demonstrated that long-term co-dependency between plants and fungal endophytes can result in the generation of a wide range of secondary metabolites, as these organisms undergo constant metabolic and environmental interactions^17,18^. Fungal root endophytes, particularly those associated with plants, present a remarkable source of biologically active natural products with potential benefits in the medicinal sector^19^. It has been widely observed that many fungal species beneficial for plant growth and development are soil-borne and are found in extreme environments such as deserts (≤ 45 °C) and mountainous regions (− 20 °C to 30 °C)^20^. This study was conducted in the transition zone or the foothills of Shivalik mountainous range of the state of Punjab, India, also known as the *Kandi* region, which comprises of dry deciduous thorn scrub forests^21^, with the aim to unearth and characterize plant root associated fungal endophytes from the natural vegetation. These roots associated microbes can be cultured axenically in a simple or complex media and may possess variety of plant growth promoting properties, such as the solubilization of Phosphorous (P), Zinc (Zn) and Iron (Fe), along with the production of important phytohormones such as auxin (Indole 3-Acetic Acid) and Gibberellic acid^22^. In this study, a polyphasic approach incorporating phylogenetic analysis of partial *ITS*, β-tubulin (*BenA*), calmodulin (*CaM*), and RNA polymerase II second-largest subunit (*RPB2*) gene sequences, along with macro- and micro-morphological data, was employed to characterize the newly discovered fungal strain.

## Materials and method

### Origin and sampling of rhizospheric soil

Rhizospheric soil samples, along with roots, from 12 plant species including *Mimosa himalayana*, *Desmodium triflorum*, *Mangifera indica*, *Geranium wallichianum*, *Crataeva nurvala*, *Terminalia arjuna*, native to the local vegetation of the adjoining forest area of Kandi region (31°10′6″N 76°28′50″E), were collected using a small wooden trowel. The samples, weighing 250–500 g, were stored in airtight zip lock bags at 4 °C for further processing in the laboratory. The isolation and screening of fungal root endophytes were carried out following the protocols^9,23^ with slight modifications.

The collected root samples were subjected to a thorough washing process. Initially, they were rinsed with running tap water to remove any attached soil debris, followed by three rinses with sterile distilled water. To ensure surface sterilization, the root segments (~ 1 cm) were treated with 70% ethanol for 45 s, then rinsed again with sterile distilled water. Subsequently, the samples were dipped in a solution of 0.1% (w/v) mercuric chloride (HgCl_2_) for 60 s, followed by another rinse with sterile distilled water. The maceration of root segments was performed using a sterile mortar and pestle. To maintain the sterility of the process, 1 ml of sterile distilled water was continuously added during maceration, resulting in a final solution volume of 10 ml. To confirm the effectiveness of the surface sterilization, some intact root segments were placed on Potato dextrose agar (PDA) plates immediately after processing. Simultaneously, serial dilutions were prepared up to 10^−4^, and 100 µl of the diluted solution from dilutions 10^−2^ and 10^−4^ were spread on PDA plates using a sterile glass spreader. The plates were then incubated at 25 °C in the dark. After 7 days of incubation, fungal growth was observed, and distinct colonies showing different colony morphology and metabolite secretion were selected using a sterile loop. These colonies were inoculated onto fresh PDA plates to obtain pure cultures, ensuring careful handling to prevent overgrowth.

### Morphological analysis

The selected isolate's macroscopic characteristics were examined on different media and under varying growth conditions. Plates containing the isolate were incubated in darkness at 25 °C for 7 days. After this incubation period, morphological traits were recorded. The culture was then inoculated onto Potato dextrose agar (PDA), malt extract agar (MEA), Czapek yeast extract agar (CYA), CYA supplemented with 5% NaCl (CYAS), creatine sucrose agar (CREA), dichloran 18% glycerol agar (DG18), and oatmeal agar (OA) in 90 mm Petri dishes^24^. The plates were again incubated in darkness at 25 °C for 7 days. After this incubation period, colony diameters were measured, and observations were made regarding sporulation, obverse and reverse colony colors, and the presence of soluble pigments. Colony colors were identified using the color codes^25^. For the assessment of ascoma production, oatmeal agar plates were incubated for a maximum of 3 weeks.

### Microscopic analysis

Confocal microscopy (CM) and scanning electron microscopy (SEM) were employed to assess the fungal ultrastructure and surface morphology. In CM, Wheat Germ Agglutinin, Alexa Fluor™ 488 Conjugate fluorescent dye (specific to fungi) was utilized to highlight spore germination and mycelial growth. Observations were made using a Nikon A1 confocal microscope at a magnification of 60× with 3× digital zoom.

To examine the ascomata and ultrastructure, the fungal mat segment (approximately 0.2 mm) consisting of spores and mycelia from a 12-day-old colony was fixed in distilled water on a small glass slide (one cm^2^ area) and observed using a Nikon A1 confocal microscope at a magnification of 60× with 3× digital zoom.

For SEM analysis, a fungal mat segment (approximately 0.2 mm) comprising spores and mycelia from a 12-day-old colony was fixed in a 2.5% glutaraldehyde solution in 0.1 M phosphate saline buffer on a small glass slide (one cm^2^ area). The fungal sample was then coated with gold within a vacuum chamber to enhance conductivity and examined at various magnifications.

### DNA extraction, PCR amplification and sequencing

Genomic DNA was extracted from a 12-day-old culture using an in-house Fungal DNA isolation kit, and its purity and concentration were determined using a Denovix DS-11 spectrophotometer. The *ITS* region, as well as regions of the *RPB2*, *BenA*, and *Cmd* genes, were amplified following the protocol^26^ with specific primers listed in supplementary Table 1. The amplification was carried out using Taq DNA Polymerase 2× Master Mix RED (Cat. No. A180301) on a GeneAmp PCR System 9700. The PCR amplicons obtained were purified using Exo-SAP purification. Bi-directional cycle sequencing was performed using forward and reverse primers with the BDT V3.1 Cycle sequencing kit on an ABI 3730 Genetic Analyzer.

### Sequence alignment and phylogenetic analysis

The raw sequences obtained in this study from four different loci were manually proof-read and edited using BioEdit 7.0.9^27^. The edited sequences were aligned using ClustalX (v2.1)^28^ and visualized using Jalview (v2.8)^29^. To ensure accurate phylogeny inference, the aligned sequences were further trimmed using GeneDoc 2.7 software. The percentage pairwise identity was computed using EBI's MUSCLE tool and presented as a Heat-Map using Morpheus software (https://software.broadinstitute.org/Morpheus). Evolutionary relationships were determined through the Maximum Likelihood (ML) method, employing the Kimura 2-parameter model. Initial tree(s) for the heuristic search were obtained by applying the Neighbor-Joining method and subjected to 500 bootstrap replications, with substitution model and rates among sites as stated in supplementary Table 2, to a matrix of pairwise distances estimated using the Maximum Composite Likelihood (MCL) approach and then selecting the topology with superior log likelihood value. The resulting tree was drawn to scale, with branch lengths representing the number of substitutions per site. Ambiguous positions were removed for each sequence pair. Evolutionary analyses were conducted using MEGA software^30^.

### Plant growth promotion assay

Fungal isolate was further assessed for its ability to pertain plant growth promoting traits such as production of IAA, phosphate, zinc, and silica solubilization.

### IAA production

IAA quantification was conducted following the protocol^31^. In this method, test tubes containing 10 ml of Czapek Dox medium (pH 6.5), with or without tryptophan supplementation, were inoculated with a HNB9 fungal agar plug (0.2 cm radius). The tubes were then placed in darkness and incubated at a temperature of 25 °C for a period of 15 days. After the incubation period, the culture filtrate was obtained by centrifuging the samples at 8000 rpm for 10 min. Next, 1 ml of the culture filtrate was mixed with two ml of Salkowski reagent and left to incubate in darkness for 5 min at room temperature. Optical density measurements of the prepared aliquots were taken at a wavelength of 530 nm using a UV/VIS spectrophotometer (Lab India, UV3000). The concentration of IAA was determined by comparing the obtained optical density values with a standard curve established using known concentrations of IAA.

### Phosphate solubilization

Phosphate solubilization was evaluated using Pikovskaya's agar medium. Cultures were inoculated onto freshly prepared Pikovskaya's agar plates using a three-point method. The plates were then incubated for 7 days at a temperature of 25 °C. The formation of a clear zone around the fungal hyphae indicated the ability to solubilize inorganic phosphorous^32^.

### Zinc solubilization

Zinc solubilization was assessed using minimal media supplemented with 0.1% zinc oxide, following the method^33^. Cultures were inoculated on the minimal media plates in a three-point pattern and incubated for 7 days at 25 °C. The presence of a clear zone around the fungal colony was observed as an indication of zinc solubilization.

### Silica solubilization

Silica solubilization was assessed using Bunt and Rovira solid medium supplemented with 0.25% magnesium trisilicate, following the method^34^. Cultures were inoculated on the agar plates in a three-point pattern and incubated for 7 days at 25 °C. Observations were made to determine the formation of a clear zone around the growing fungal colony, indicating silica solubilization.

### Assessment of fungal–plant interaction

The initial screening involved evaluating colony morphology and metabolite production of twelve fungal isolates. In-vitro plant–microbe interaction was conducted to further analyze these isolates. Surface sterilization of mustard seeds (*Brassica juncea* var. Varuna) was performed using a five % sodium hypochlorite (NaOCl) solution supplemented with 0.2% Tween20™ (one drop for 100 ml NaOCl), followed by rinsing with sterile distilled water. The seeds were then immersed in 70% ethanol for 40–50 s and washed again with distilled water. Specialized screw cap jam bottles (autoclavable) with 350 ml capacity were prepared, containing 100 ml of Murashige and Skoog media^35^ slants at half strength. The surface-sterilized seeds were carefully placed on top of the slants. A fungal agar plug (0.2 cm) of the selected isolate was placed at the bottom of the slants, which were then kept in the dark at a temperature of 24 ± 2 °C. After the roots emerged (within 24–48 h), the slants were transferred to a plant tissue culture facility with a 16:8 h photoperiod (light intensity > 3500 Lux) to monitor the plant–microbe interaction. After 15 days of inoculation, the slants were evaluated to determine the pathogenicity of the fungal isolate towards the host plant.

### Pot trials

For pot trials, a stock solution of 0.1% saline solution (NaCl) containing 2.8 × 10^6^/ml fungal spores were prepared. The soil mixture in an equal ratio of sterilized compost, garden soil, and vermiculite was subjected to autoclaving for three consecutive days, each lasting 40 min at a pressure of 1.034 × 10^5^ Pa and a temperature of 121 °C. Pots containing 500 g of soil mixture were prepared. Seeds were sown in the pots, and a treatment of 100 µl of the stock solution was applied. Inside the greenhouse, a relative humidity of 75% was maintained, along with a light intensity of 12,000 Lux and a photoperiod of 16:8 h. Daily watering of all pots was carried out using tap water, providing 45% of the maximum water holding capacity of the pots.

### Assessment of root colonization

Root samples from plants that interacted with the fungal isolate both in-vitro and in vivo were carefully extracted using sterile forceps. The extracted roots were immersed in sterile distilled water at a lukewarm temperature of around 40 °C to eliminate any residue attached to the root surface. Assessment of root colonization was conducted following the protocol^9^. The distribution of chlamydospores was used as an indicator of colonization, and observations were made using a digital Nikon Eclipse E 300 microscope at a magnification of 40×.

### Statistical analysis

All experiments in this study were performed in triplicate, and the results are presented as the average ± SD. Statistical analysis was conducted using STATISTICA 10.0 software (StatSoft Inc., USA). The effects of each treatment were assessed using one-way analysis of variance (ANOVA), and pairwise comparisons among the means were determined by calculating the least significant difference (LSD) with a significance level of p ≤ 0.05.

### Declaration

All methods were carried out in accordance with relevant guidelines.

## Results

The majority of isolated fungal colonies were identified as belonging to *Aspergillus*, *Fusarium*, and *Trichoderma* species based on their morphological and microscopic characteristics. Notably, one of the mixed colonies, named HNB9, exhibited a unique red color and showed highly promising results during primary in-vitro interaction studies with Mustard.

### Macroscopic analysis of *T. albobiverticillius* HNB9

**Figure 1:**
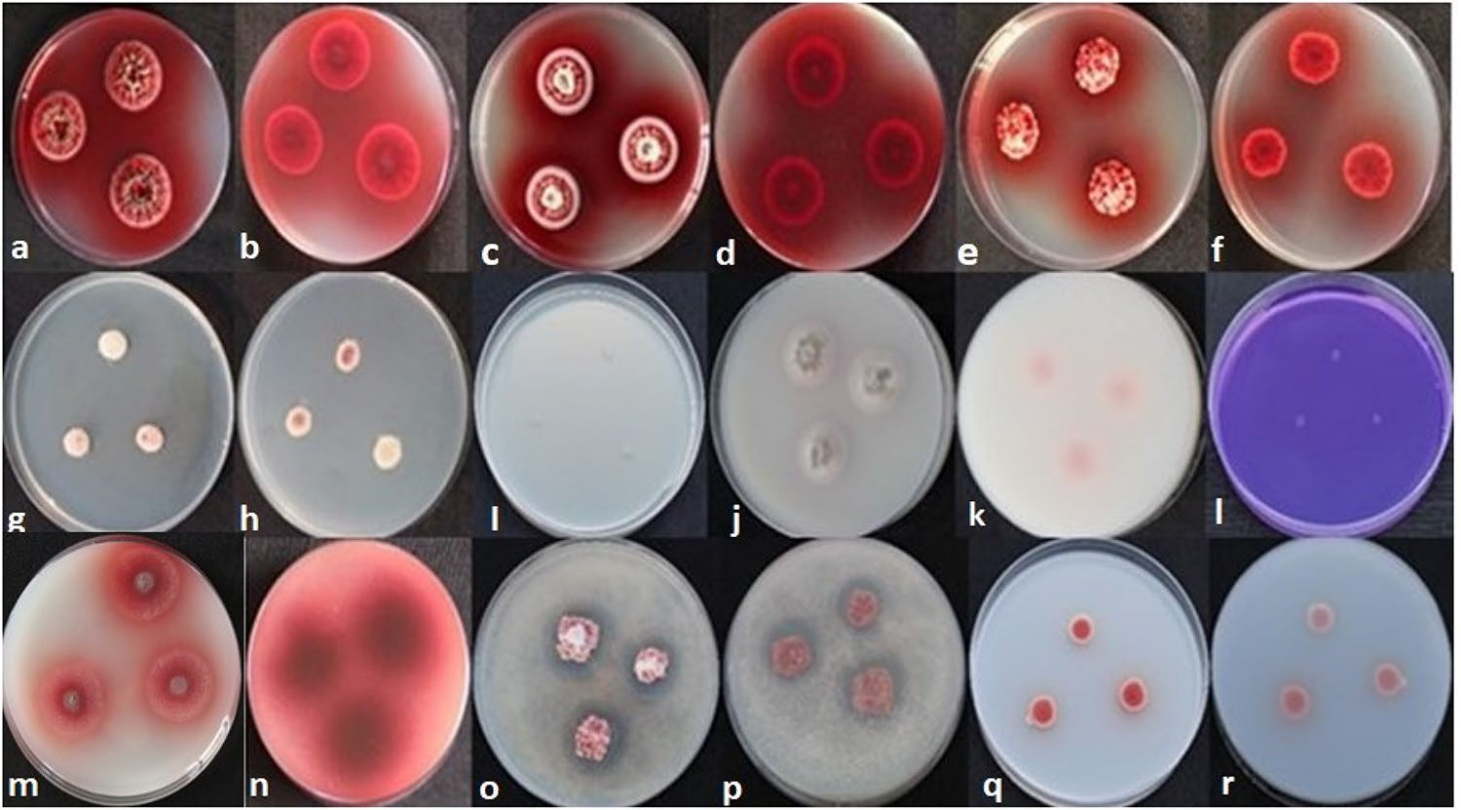
*T*. *albobiverticillius* HNB9 on PDA (**a**,**b**), MEA (**c**,**d**), CYA (**e**,**f**), DG18 (**g**,**h**), CYAS (**i**), OMA (**j**,**k**), CREA (**l**) and solubilization of phosphate (**m**,**n**), zinc (**o**,**p**) and silica (**q**,**r**). Colony obverse and reverse are shown in every media except CYAS and CREA (obverse).

The macroscopic characteristics of the selected fungal isolate were examined under various growth conditions and on different media (Fig. 1; Table 1). On potato dextrose agar (PDA) at 25 °C for 7 days, the colonies measured 24–30 mm in diameter. They displayed white mycelia, with floccose mycelia present at the center. Exudate and soluble pigment were observed, indicating a luscious growth. Abundant green conidia were also present. The surface color ranged from white to red, while the reverse color appeared reddish brown with distinct radial furrow zonation, accompanied by dense sporulation.

**Table 1:** Morphological characteristics of the fungal isolate *T*. *albobiverticillius* HNB9.

Morphological features	Morphological parameters	Culture media (fungal culture has been grown for 7 days at a temperature of 25 °C)						
		PDA	MEA	CYA	DG18	CYAS	OMA	CREA
Colony morphology	Size	24–30 mm	22–26 mm	22–26 mm	8–12 mm	No growth	15–18 mm	< 1 mm
	Surface color	White to red	White to red	White to red	White to red	N/A	White to green	White
	Reverse color	Reddish brown with radially furrow zonation	Reddish brown with concentric rings	Red with radially furrow zonation	White to brown with radial zonation	N/A	White to brown with radially furrow zonation	White
Hyphal characteristics	Sporulation	Dense	Dense	Moderate	Poor	N/A	Low	Not present
	Exudate	Present	Present (Red droplets)	Present	Absent	N/A	Absent	N/A
	Pigment	Present	N/A	Present	Present	N/A	Present	N/A
	Mycelia colour	White	White	White	White	N/A	White	N/A
	Mycelia texture	Floccose (at centre)	Velvety overlaying floccose	N/A	Floccose (at centre)	N/A	N/A	N/A
	Mycelia growth	Yes	N/A	Yes	Restricted	N/A	Restricted growth of mycelia	Poor growth
Conidial morphology	Color	Green (abundance)	Green	Yellow	N/A	N/A	Green	N/A

N/A designates data not available.

On malt extract agar (MEA) at 25 °C for 7 days, the colonies measured 22–26 mm in diameter and exhibited a pinkish color due to the diffusion of exudates into the mycelia. The mycelia appeared white with a velvety texture overlaying floccose mycelia. Green conidia and red droplets of exudate were observed. Similar to the PDA, the surface color ranged from white to red, with a reddish-brown reverse color and the presence of concentric rings and dense sporulation.

On Czapek yeast extract agar (CYA) at 25 °C for 7 days, the colonies measured 22–26 mm in diameter. The mycelia were white, exhibiting moderate sporulation. Yellow conidia, exudate, and soluble pigment were present, indicating luscious growth. The surface color ranged from white to red, and the reverse color appeared red with radial furrow zonation.

On Dichloran 18% Glycerol Agar (DG 18) at 25 °C for 7 days, the colonies measured 8–12 mm in diameter. White mycelia were observed, with floccose mycelia present at the center. However, no exudate was detected. A soluble pigment was present, and the growth was restricted. The surface color varied from white to red, while the reverse color ranged from white to brown with radial zonation and poor sporulation.

No growth was observed on CYA supplemented with five % NaCl (CYAS) at 25 °C for 7 days.

On Oatmeal Agar (OMA) at 25 °C for 7 days, the colonies measured 15–18 mm in diameter and exhibited white mycelia. Sporulation was low, and the conidia appeared green. No exudate was observed, but a soluble pigment was present and the mycelial growth was restricted. The surface color ranged from white to green, and the reverse color varied from white to brown with radially furrow zonation.

On Creatine Agar (CREA) at 25 °C for 7 days, the colonies were less than 1 mm in diameter. Both the surface and reverse color were white. The growth was poor, and no sporulation occurred.

### Microscopic analysis of *T. albobiverticillius* HNB9 ultrastructure

**Figure 2:**
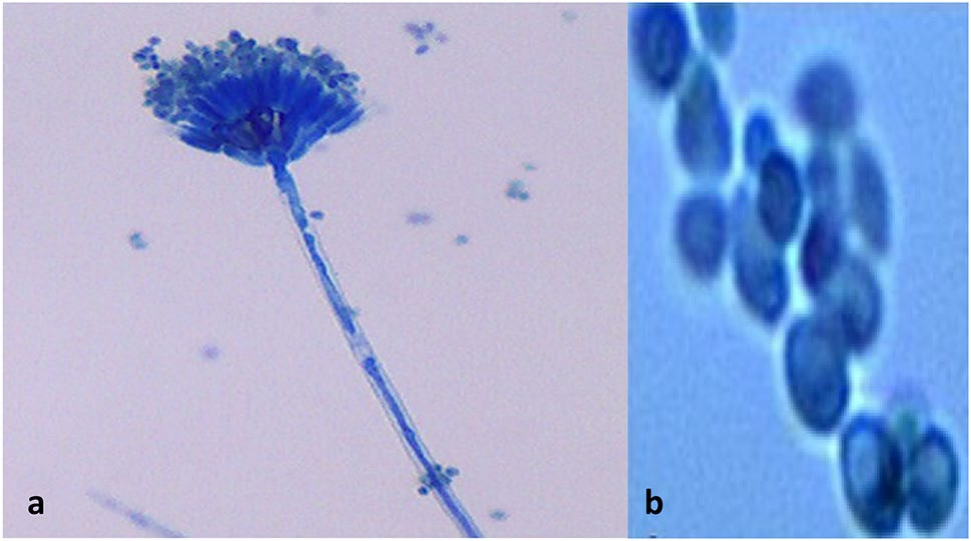
Light microscopy of *T*. *albobiverticillius* HNB9. (**a**) Biverticillate conidiophores bearing numerous asexual conidia, and septate hyphae at ×40 magnification stained with lacto phenol cotton blue. (**b**) Spores appeared to be globose to ellipsoidal at ×100 magnification.

Under light microscopy, examination of HNB9 revealed long filamentous and septate hyphae, as well as biverticillate conidiophores that bore numerous asexual conidia when viewed at 40× magnification stained with lacto phenol cotton blue (Fig. 2a). At 100× magnification, the spores appeared globose to ellipsoidal (Fig. 2b).

**Figure 3:**
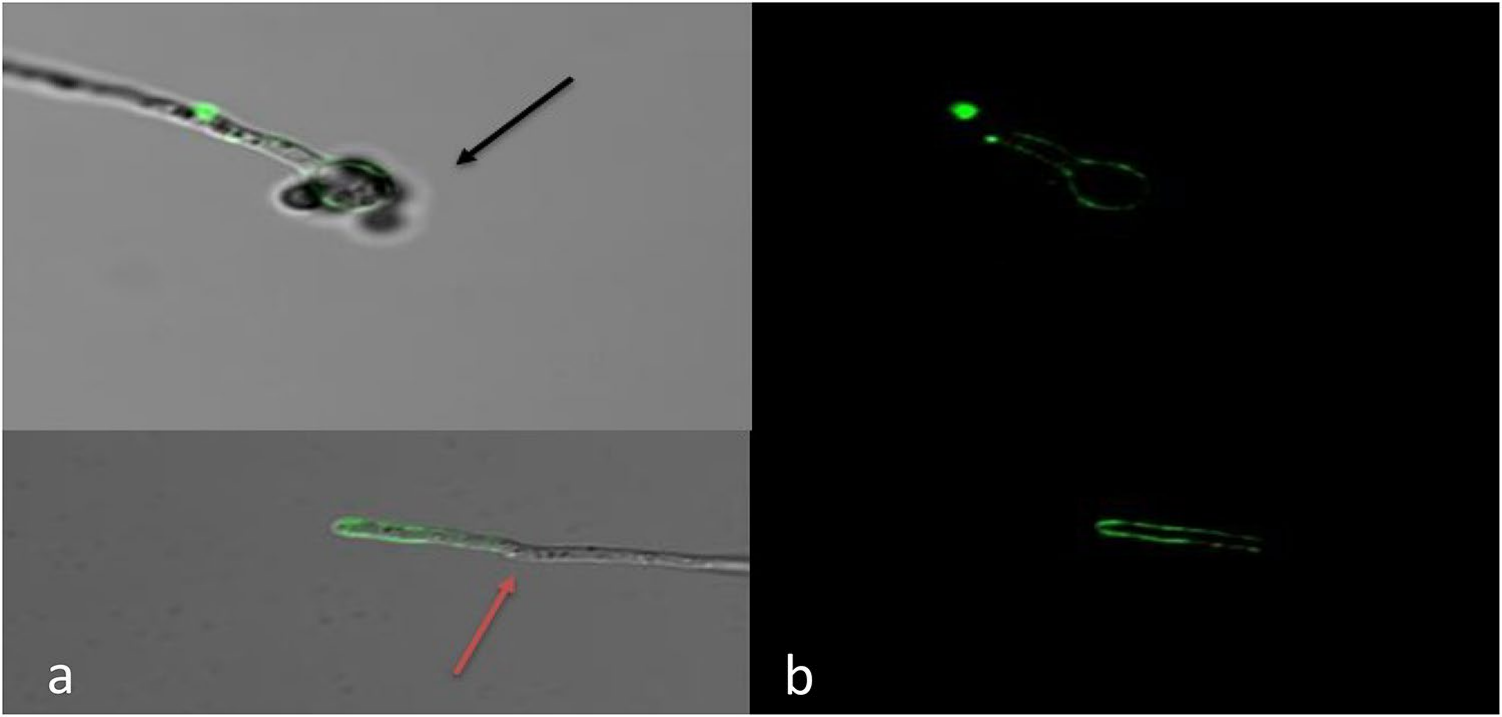
Confocal microscopy of *T. albobiverticillius* HNB9 spores (black arrow) and mycelia (red arrow); (**a**) Bright field/transmitter detector, (**b**) WGA Alexa fluor 488 dye (×60 magnification + 3× digital zoom).

Confocal images of *T. albobiverticillius* HNB9 stained with fungal-specific WGA *Alexa Fluor* 488 dye provided further insight, showing the formation of germ tubes from the spores (Fig. 3).

**Figure 4:**
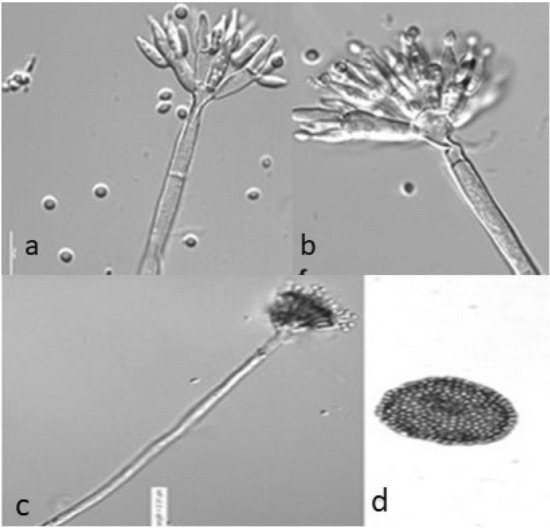
Confocal images of morphological features of *T*. *albobiverticillius* HNB9 (scale bar 10 µm). (**a**) Conidia were globose to ellipsoidal and smooth-walled (2.41 μm wide). (**b**) Stipes are smooth walled (158.8–160 × 4.18–4.2 μm), metulae are 9–15 per stipe (9.09 μm), phialides (8.8 μm) are 6–7 per metula. (**c**) Conidiophores are strictly biverticillate with broad penicillia typically measuring 115–500 µm in length hosting numerous asexual conidia. (**d**) It shows the presence of ascomata (40.38 μm) and ascospores (2.05 μm).

Assessment of ultrastructure using a Confocal Microscope revealed that conidiophores originated from surface hyphae with stipes. The conidia were globose to ellipsoidal and had smooth walls, measuring 2.41 μm in width (Fig. 4a). The stipes exhibited a smooth surface with dimensions of 158.8–160 × 4.18–4.2 μm. Each stipe had 9–15 metulae, with an average size of 9.09 μm (Fig. 4b). Phialides, measuring 8.8 μm, were found in 6–7 per metula (Fig. 4b). The conidiophores displayed a strict biverticillate pattern, with broad penicillia typically ranging from 115 to 500 µm in length and hosting numerous asexual conidia (Fig. 4c). Additionally, the presence of ascomata (40.38 μm) and ascospores (2.05 μm) was observed (Fig. 4d).

**Figure 5:**
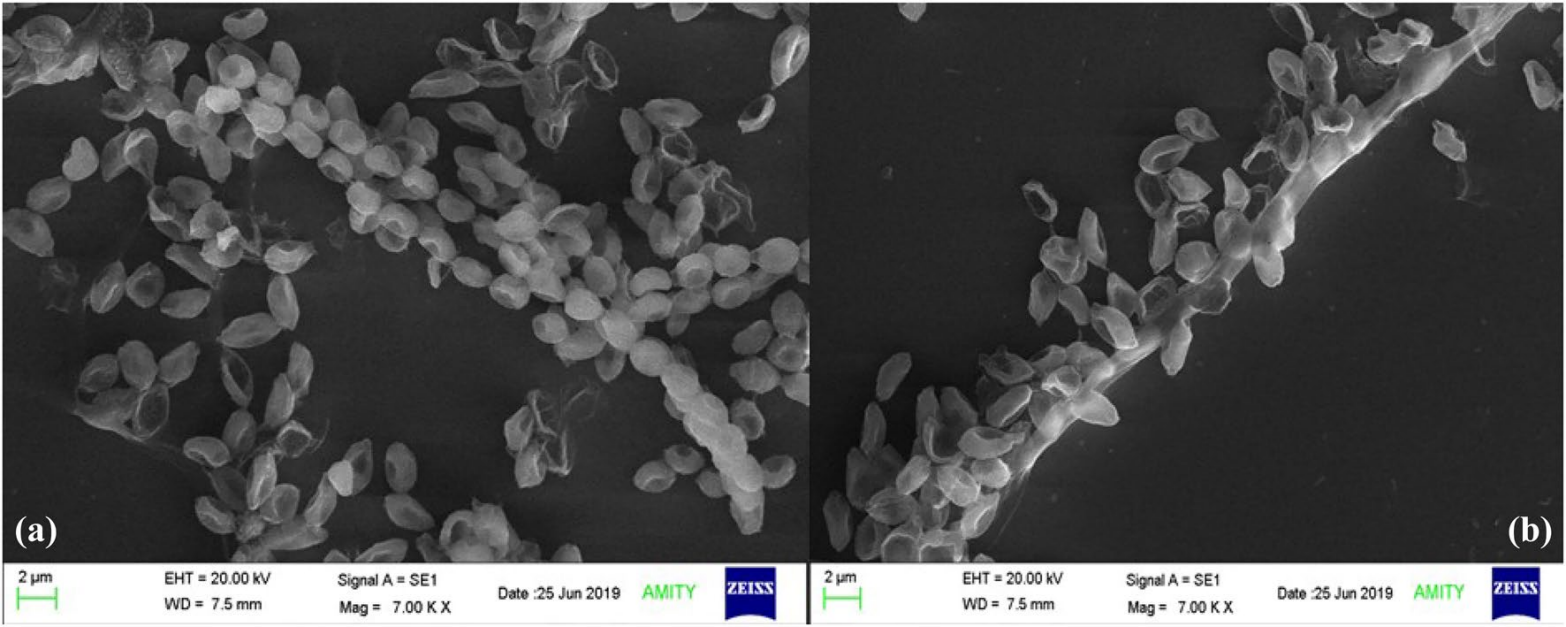
Scanning electron microscopy of *T. albobiverticillius* HNB9; (**a**) Spores produced in chain; (**b**) HNB9 mycelia surrounded with numerous spores.

Scanning electron microscopy of *T. albobiverticillius* HNB9 provided a closer look at the spores, which exhibited a smooth surface and varied in size between 2 and 4 µm (Fig. 5). The ultra-structures of the HNB9 conidia, as revealed by scanning electron microscopy, were spherical, with a diameter of 2.5–3.5 µm and a smooth or slightly rough surface (Fig. 5). The spores were also observed to be sickle-shaped and produced in chains.

### Phylogeny and pairwise sequence comparison of *T. albobiverticillius* HNB9

This study explores the phylogenetic relationships and pairwise sequence comparisons of the *Talaromyces albobiverticillius* HNB9 isolate in comparison to 35 other *Talaromyces* species, categorized into sect. *Talaromyces* (11 species) and sect. *Trachyspermi* (24 species) (Table 2). The analysis focuses on the *ITS*, *BenA*, *CaM*, and *RPB2* loci, providing valuable insights into the evolutionary relationships among these fungal species. The individual *ITS*, *BenA*, *CaM* and *RPB2* datasets consist of 857, 501, 519 and 1004 characters, respectively (Table 3) and were used to study the relationship within *Talaromyces*.

**Table 2:** Nucleotide composition of the fungal isolate *T*. *albobiverticillius* HNB9 at four different loci.

S. no.	Seq_ID	Sequence length (bp)	A Mol%	T Mol%	C Mol%	G Mol%	G + C content (%)	A + T content (%)
1	HNB9_*ITS*_contig	857	186 21.70	197 22.99	222 25.90	252 29.40	55.30	44.69
2	HNB9_*BenA*_contig	501	133 26.55	125 24.95	126 25.15	117 23.35	48.50	51.50
3	HNB9_*CaM*_contig	519	143 27.55	125 24.08	126 24.28	125 24.08	48.36	51.63
4	HNB9_*RPB2*_contig	1004	252 25.1	257 25.6	239 23.8	256 25.5	49.30	50.70

**Table 3:** Species, strains and their corresponding GenBank accession numbers of sequences used for phylogenetic analyses.

S. no.	Species	Strain/isolate	Origin	Substrate	GeneBank accessions			
					*ITS*	*BenA*	*CaM*	*RPB2*
1	*T. solicola*	DAOM 241015	South Africa	Soil	*	*	KJ885279.1	KM023295.1
		Pen193	South Africa	Soil	FJ160264.1	*	*	*
		CV191	South Africa	Soil	*	GU385731.1	*	*
2	*T. calidicanius*	CBS 112002	China: Taiwan	Soil	JN899319.1	HQ156944.1	KF741934.1	KM023311.1
3	*T. assiutensis*	CBS 147.78	Egypt	Soil	JN899323.1	KJ865720.1	KJ885260.1	KM023305.1
4	*T. minioluteus*	CBS 642.68	Unknown	Unknown	JN899346.1	MN969409.1	KJ885273.1	JF417443.1
5	*T. udagawae*	CBS 579.72	Japan	Soil	JN899350.1	KF114796.1	KX961260.1	*
		DTO 302-A8	Japan	Soil	*	*	*	MN969148.1
6	*T. erythromellis*	CBS 644.80	Australia	Soil	JN899383.1	HQ156945.1	KJ885270.1	KM023290.1
7	*T. diversus*	CBS 320.48	USA	Mouldy leather	KJ865740.1	KJ865723.1	KJ885268.1	KM023285.1
8	*T. rubrifaciens*	CGMCC:3.17658	China: Beijing	Hospital air	KR855658.1	KR855648.1	KR855653.1	KR855663.1
9	*T. aerius*	CBS 140611	China: Beijing	Indoor air	KU866647.1	*	*	KU866991.1
		DTO 317-C7	China: Beijing	Indoor air	*	KU866835.1	KU866731.1	*
10	*T. heiheensis*	HMAS 248789	China: Heilongjiang	Rotten wood	KX447526.1	KX447525.1	KX447532.1	KX447529.1
11	*T. minnesotensis*	DI16-144	USA	Human ear	LT558966.1	LT559083.1	LT795604.1	LT795605.1
12	*T. catalonicus*	FMR 16441	Spain	Herbivore dung	LT899793.1	LT898318.1	LT899775.1	LT899811.1
13	*T. amyrossmaniae*	NFCCI:1919	India	Decaying fruits of *Terminalia bellerica*	MH909062.1	MH909064.1	MH909068.1	MH909066.1
14	*T. clemensii*	PPRI 26753	South Africa	Wood in mine	MK951940.1	MK951833.1	MK951906.1	MN418451.1
15	*T. guatemalensis*	CCF 6215	Guatemala	Soil	MN322789.1	MN329687.1	MN329688.1	MN329689.1
16	*T. albisclerotius*	CBS 141839	China: Guizhou	Soil	MN864276.1	MN863345.1	MN863322.1	MN863334.1
17	*T. chongqingensis*	CS26-67	China: Chongqing	Soil	MZ358001.1	MZ361343.1	MZ361350.1	MZ361357.1
18	*T. purpureogenus*	CBS 286.36	Unknown	Unknown	JN899372.1	*	KF741947.1	JX315709.1
		KAS3773	South Africa	Soil	*	JF910281.1	*	*
19	*T. albobiverticillius*	CBS 133440	China: Taiwan	Decaying leaves	*	*	KJ885258.1	KM023310.1
		900890701	China: Taiwan	Decaying leaves	HQ605705.1	*	*	*
		DTO_270B8	Thailand	Indoor house dust	*	KJ775225.1	*	*
20	*T. convolutes*	CBS 100537	Nepal	Soil	NR_137157.1	KF114773.1	MN969316.1	JN121414.1
21	*T. austrocalifornicus*	CBS 644.95	USA	Soil	*	KJ865732.1	KJ885261.1	MN969147.1
		S3D	Iran	Organic sediment complexes	MW897776.1	*	*	*
22	*T. viridulus*	CBS 252.87	Australia	Soil	JN899314.1	JX091385.1	KF741943.1	*
23	*T. liani*	CBS 225.66	China	Soil	JN899395.1	JX091380.1	KJ885257.1	*
24	*T. alveolaris*	DI16-147	USA	Human bron choalveolar lavage	LT558969.1	LT559086.1	LT795596.1	*
25	*T. muroii*	CBS _756.96	China: Taiwan	Soil	MN431394.1	KJ865727.1	KJ885274.1	*
26	*T. striatoconidius*	CBS _550.89	Cuba	Leaf litter of *Pachyanthus poirettii*	MN431418.1	MN969441.1	MN969360.1	*
27	*T. duclauxii*	CBS 322.48	France	Canvas	JN899342.1	JX091384.1	*	JN121491.1
28	*T. subericola*	FMR:15656	Spain	Sparkling wine	LT985888.1	*	LT985904.1	LT985909.1
29	*T. solicola*	Unknown	Brazil	Unknown	*	LR535945.1	LR535946.1	LR535948.1
30	*T. chongqingensis*	CS26-63	China: Chongqing	Soil	*	MZ361344.1	MZ361351.1	MZ361358.1
31	*T. chongqingensis*	CS26-73	China: Chongqing	Soil	*	MZ361345.1	MZ361352.1	MZ361359.1
32	*T. chongqingensis*	CS26-75	China: Chongqing	Soil	*	MZ361346.1	MZ361353.1	MZ361360.1
33	*T. brasiliensis*	URM 7618	Brazil	Honey	MF278323.1	LT855560.1	LT855563.1	MN969198.1
34	*T. systylus*	Unknown	Argentina	Soil	KP026917.1	KR233838.1	KR233837.1	*
35	*T. speluncarum*	FMR:16671	Spain	Culture from holotype of *Talaromyces speluncarum*	LT985890.1	*	LT985906.1	LT985911.1
36	*T. atroroseus*	CBS133442	South Africa	House dust	*	KF114789.1	KJ775418.1	KM023288.1
		DTO 390-I4	Nigeria	Unknown	MN788119.1	*	*	*
37	*T. mycothecae*	URM 7622	Brazil	Nest of stingless bee (*Melipona scutellaris*)	MF278326.1	LT855561.1	LT855564.1	LT855567.1
38	*T. stipitatus*	CBS:375.48	Netherlands	Culture from isotype of *Talaromyces stipitatus*	JN899348.1	KM111288.1	KF741957.1	KM023280.1
39	*T. verruculosus*	NRRL1050	Netherlands	Culture from neotype of *Penicillium verruculosum*	KF741994.1	KF741928.1	*	*
		CBS:254.56	Netherlands	Unknown	*	*	KF741944.1	*
		AX2101 I	Brazil	Metallic surface exposed to water	*	*	*	KJ476428.1

* Data not available for corresponding strain / isolate.

**Figure 6:**
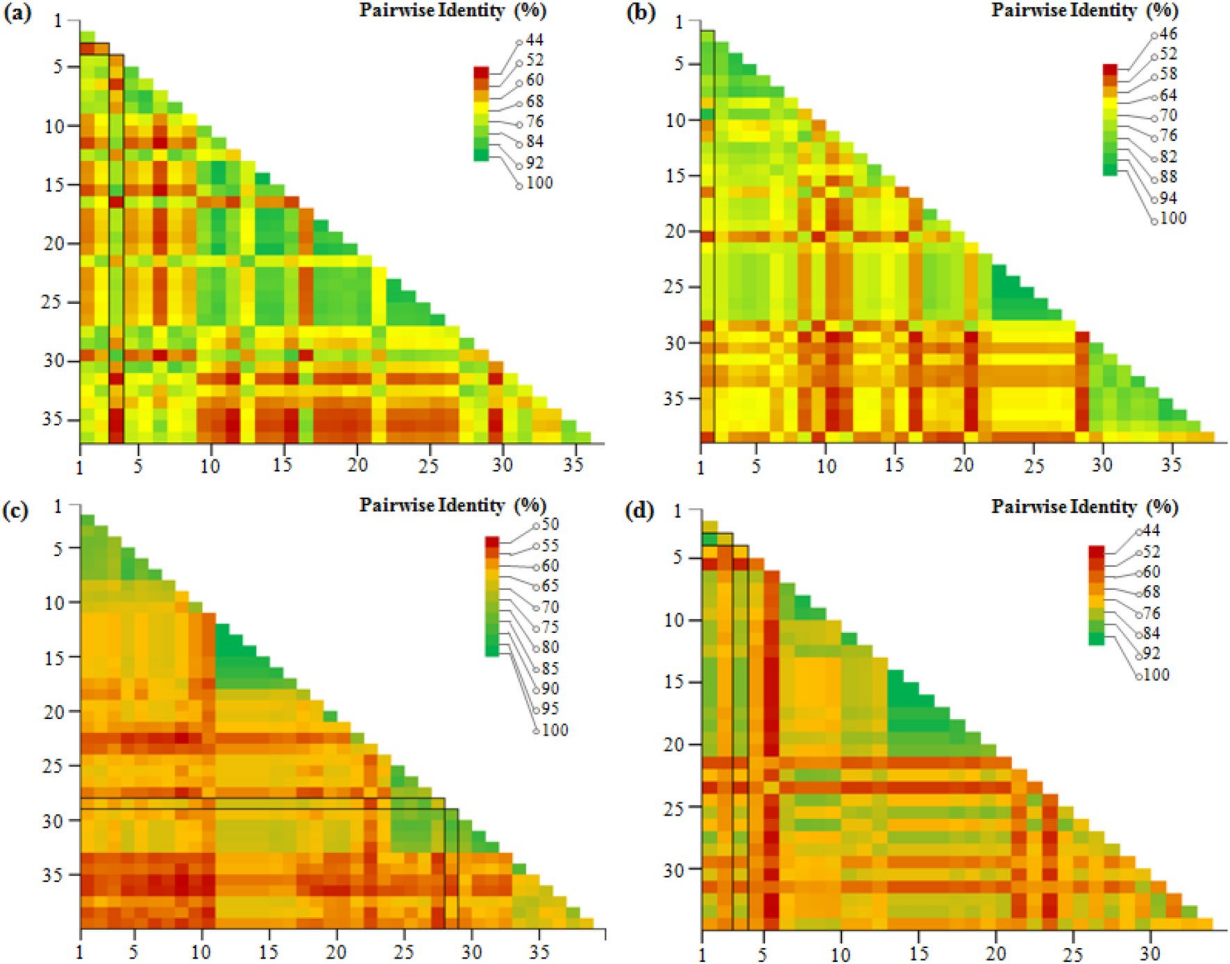
Pairwise identity matrix based on the fungal isolate HNB9 multigene sequence. A colour-coded pairwise identity matrix generated from partial (**a**) nuclear rDNA internal transcribed spacer region (*ITS*), (**b**) *BenA* (*β-tubulin*) (**c**) *CaM* (*calmodulin*) and (**d**) *RPB2* (RNA polymerase II second largest subunit) gene sequences. Each coloured cell represents the percentage identity score between two sequences. A colour key indicates the correspondence between pairwise identities and the colours displayed in the matrix. Black colour bordered box in each plot indicates the fungal isolate HNB9 used in this study and the corresponding published sequences of selected fungal species. Values on both the axes in each plot represent the fungal species and strain ID used for the comparative analysis (supplementary Tables 3–6).

Table 4 and Fig. 6 present pairwise identity data for *ITS*, *BenA*, *CaM*, and *RPB2* loci. For *ITS*, *T*. *rubrifaciens* exhibited 100% identity, while *T. albobiverticillius* and *T. heiheensis* showed high identities of 98.9% and 98.2%, respectively. In the *BenA* locus, *T. albobiverticillius* displayed the highest identity (99.77%), with *T. rubrifaciens* and *T. erythromellis* close behind. *CaM* locus identities ranged from 50 to 100%, with *T. rubrifaciens* showing the highest (97.73%). *RPB2* locus identities varied from 43 to 100%, with *T. rubrifaciens* leading at 99.11%, followed by *T. albobiverticillius*.

**Table 4:** Nucleotide identity of the sequences used under this study based on the *ITS, BenA, CaM* and *RPB2* genes.

Locus name	Pairwise identity range (%) (all possible combinations)	Pairwise identity (%) with respect to sequence used in this study (BLAST analysis)	
*ITS*	0.44–0.98 (Fig. 6a)	*T*. *rubrifaciens* (100%)	Supplementary Table 3
		*T*. *albobiverticillius* (98.9%)	
		*T*. *heiheensis* (98.2%)	
*BenA*	0.46–1.00 (Fig. 6b)	*T*. *albobiverticillius* (99.77%)	Supplementary Table 4
		*T*. *rubrifaciens* (98.99%)	
		*T*. *erythromellis* (96.4%)	
*CaM*	0.50–1.00 (Fig. 6c)	*T*. *rubrifaciens* (97.73%)	Supplementary Table 5
		*T*. *heiheensis* (93.57%)	
		*T*. *albobiverticillius* (93.44%)	
		*T*. *amyrossmaniae* (93.37%)	
*RPB2*	0.43–1.00 (Fig. 6d)	*T*. *rubrifaciens* (99.11%)	Supplementary Table 6
		*T*. *albobiverticillius* (98.47%)	
		*T*. *erythromellis* (98%)	
		*T*. *heiheensis* (97.31%)	
		*T*. *catalonicus* (97.23%)

**Figure 7:**
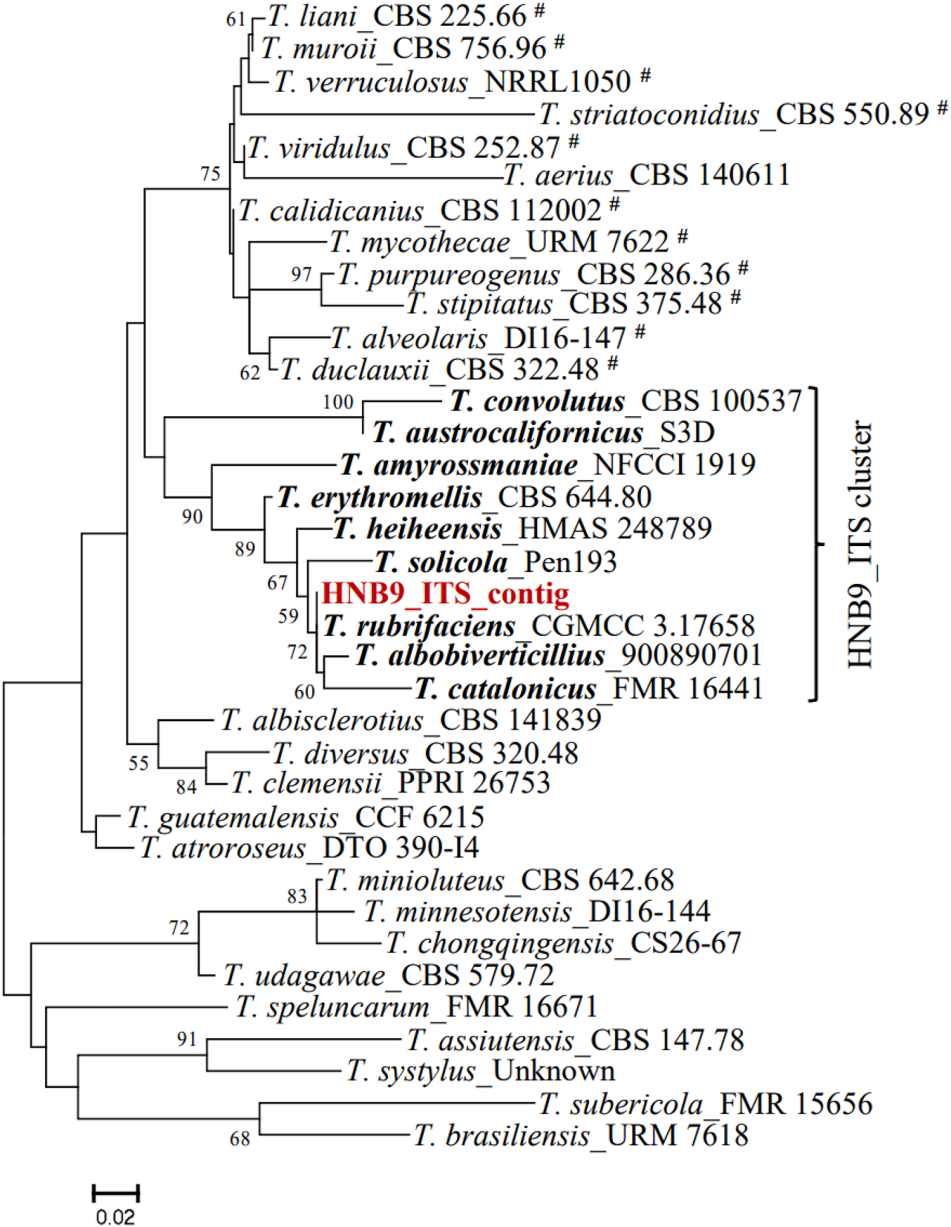
Molecular Phylogenetic analysis by Maximum Likelihood method inferred from partial *ITS* sequences. Bootstrap percentages ≥ 50% derived from 500 replicates are indicated at the nodes. The bar indicates the number of substitutions per position. The sequence used in this study was shown in dark red colour. Cluster specific accessions were highlighted in bold. #: sect. *Talaromyces* and the remaining belong to sect. *Trachyspermi*.

The most optimal model for each loci used in phylogeny is listed in Supplementary Table 2. The phylogenetic tree (Fig. 7) for the *ITS* locus reveals that HNB9_ITS_contig is closely related to *T. rubrifaciens*_CGMCC 3.17658 and *T_albobiverticillius*_900890701, forming a distinct branch connected to *T. catalonicus*_FMR 16441. This suggests a recent common ancestor, with closer relatedness to *T*. *rubrifaciens*_CGMCC 3.17658 and *T. albobiverticillius*_900890701.

**Figure 8:**
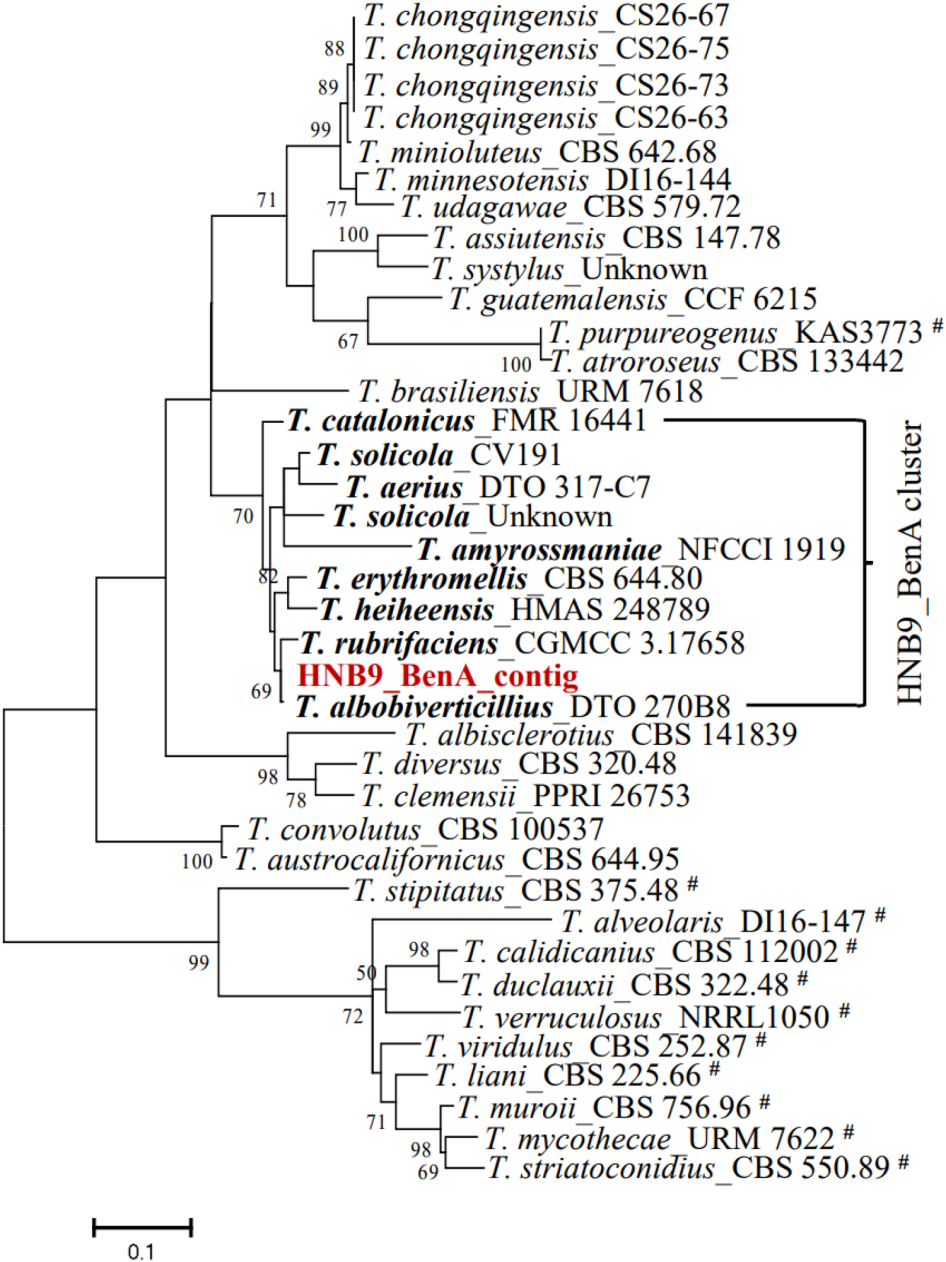
Molecular Phylogenetic analysis by Maximum Likelihood method inferred from partial *BenA* sequences. Bootstrap percentages ≥ 50% derived from 500 replicates are indicated at the nodes. The bar indicates the number of substitutions per position. Sequence used in this study was shown in dark red colour. Cluster specific accessions were highlighted in bold. #: sect. *Talaromyces* and the remaining belongs to sect. *Trachyspermi*.

In Fig. 8, the phylogenetic tree for the *BenA* locus places HNB9_BenA_contig with *T*. *rubrifaciens*_CGMCC 3.17658 and *T. albobiverticillius*_DTO_270B8 in a subclade. The branching pattern indicates a more recent common ancestor with *T. rubrifaciens*_CGMCC 3.17658, suggesting a close relationship.

**Figure 9:**
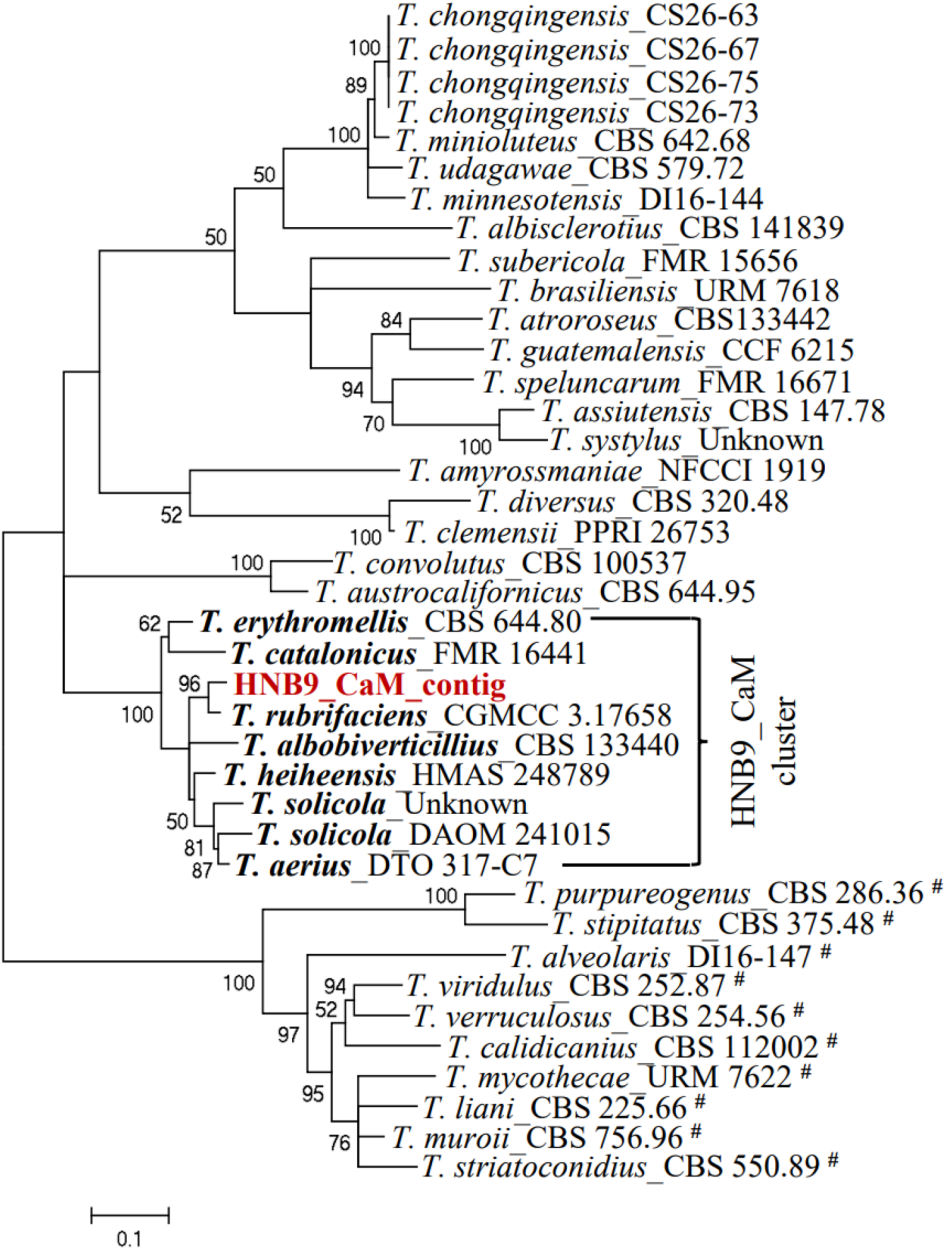
Molecular Phylogenetic analysis by Maximum Likelihood method inferred from partial *CaM* sequences. Bootstrap percentages ≥ 50% derived from 500 replicates are indicated at the nodes. The bar indicates the number of substitutions per position. Sequence used in this study was shown in dark red colour. Cluster specific accessions were highlighted in bold. #: sect. *Talaromyces* and the remaining belong to sect. *Trachyspermi*.

Figure 9 illustrates the phylogenetic tree for the *CaM* locus, grouping HNB9_CaM_contig with *T*. r*ubrifaciens*_CGMCC 3.17658 under a common branch, indicating a relatively recent common ancestor.

**Figure 10:**
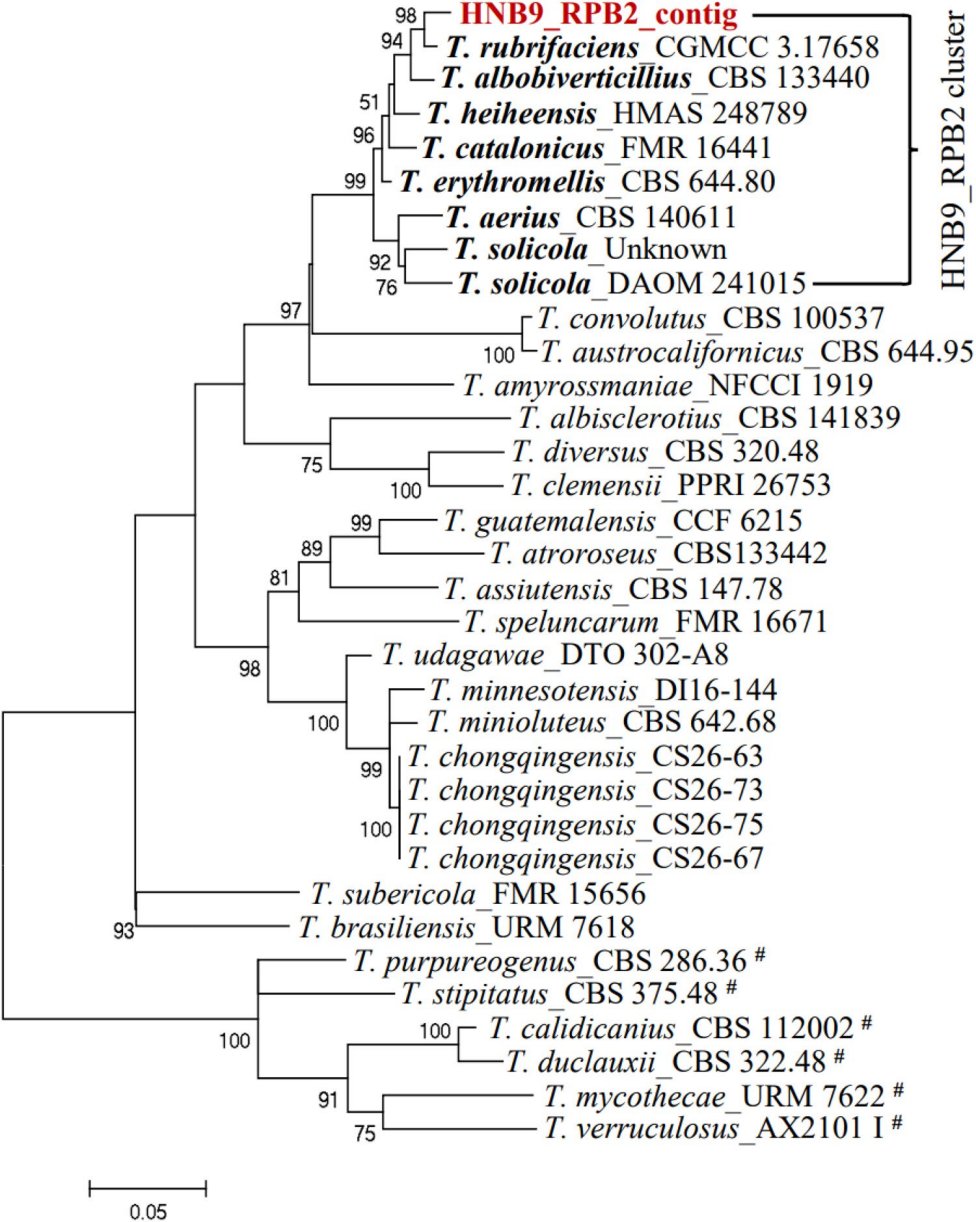
Molecular Phylogenetic analysis by Maximum Likelihood method inferred from partial *RPB2* sequences. Bootstrap percentages ≥ 50% derived from 500 replicates are indicated at the nodes. The bar indicates the number of substitutions per position. The sequence used in this study was shown in dark red colour. Cluster specific accessions were highlighted in bold. #: sect. *Talaromyces* and the remaining belong to sect. *Trachyspermi*.

In Fig. 10, the phylogenetic tree for the *RPB2* locus groups HNB9_RPB2_contig with *T*. *rubrifaciens*_CGMCC 3.17658 and *T. albobiverticillius*_CBS 133440, forming a distinct clade. The short branch length suggests a closer genetic relationship between HNB9_RPB2_contig and *T. rubrifaciens*_CGMCC 3.17658.

The sequence data obtained from the study has been submitted to the National Center for Biotechnology Information (NCBI), and the corresponding accession numbers for the sequences are as follows:

- HNB9_*BenA*_contig: ON406962
- HNB9_*CaM*_contig: ON406963
- HNB9_*RPB2*_contig: ON406964
- HNB9_*ITS*_contig: ON261679

Furthermore, the type culture associated with the study has been submitted to the National Agriculturally Important Microbial Culture Collection (NAIMCC), which is part of the ICAR-National Bureau of Agriculturally Important Microorganisms (NBAIM) *Kushmaur*, *Mau Nath Bhanjan* Uttar Pradesh, India. The accession number assigned to the type culture is NAIMCC-SF-0025.

These accession numbers and the submission to NAIMCC provide a standardized reference for accessing and referencing the specific genetic sequences and type culture associated with the study.

### Plant growth promoting (PGP) properties

**Figure 11:**
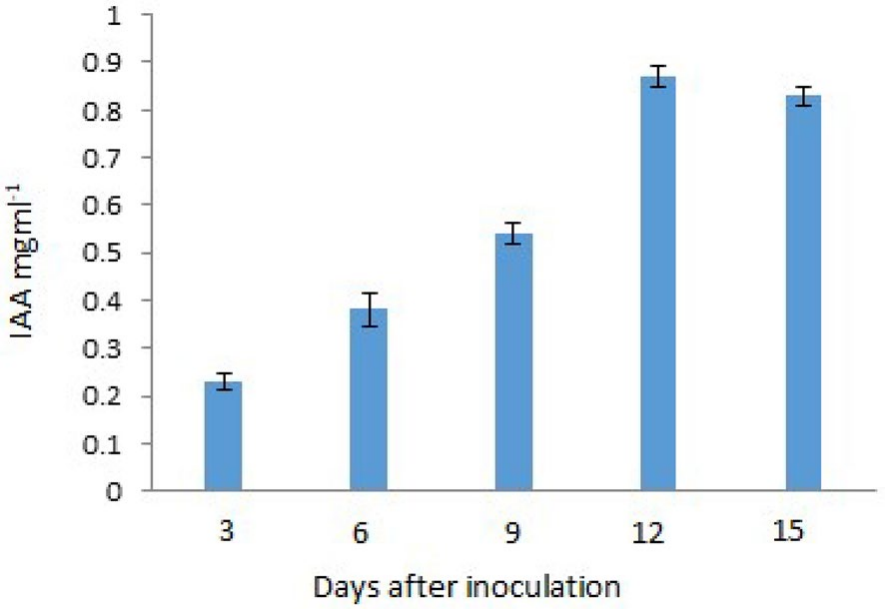
Maximum production of IAA was observed to be 0.85 ± 0.02 and 0.81 ± 0.02 mg/l after 12 and 15 days of incubation respectively.

*T. albobiverticillius* HNB9 was found to colonize plant roots and exhibit plant growth-promoting properties, leading to enhanced plant growth and development. These properties include the solubilization of zinc, phosphorus, and silica, and the production of indole-3-acetic acid (IAA), a plant growth-regulating hormone. The solubilization index for silica was determined to be 2.33, while for zinc and phosphorus, it was consistently measured at 2.33 and 2.58, respectively (Fig. 1). Furthermore, the production of IAA by *T. albobiverticillius* HNB9 was quantified, and the maximum IAA production was observed to be 0.85 ± 0.02 mg/L after 12 days of incubation, with a similar level of production (0.81 ± 0.02 mg/L) maintained after 15 days of incubation (Fig. 11). These findings highlight the plant growth-promoting potential of *T. albobiverticillius* HNB9, which contributes to its ability to enhance plant growth and development.

### Assessment of fungal–plant interaction

**Figure 12:**
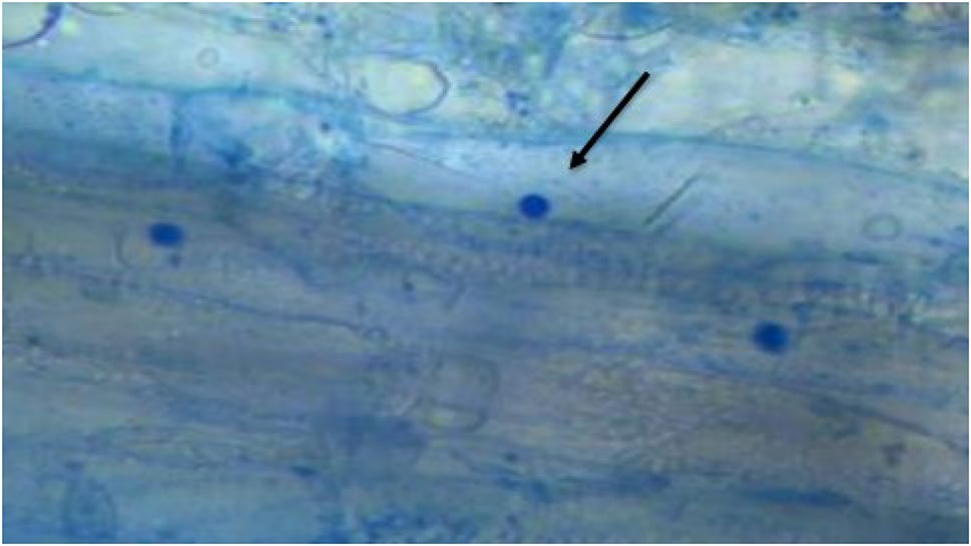
*T. albobiverticillius* HNB9 spores colonizing roots of host plant (Okra) stained with Lacto phenol cotton blue dye (×40 magnification).

Our *in-vitro* and greenhouse studies involving mustard, maize, and okra revealed significant benefits of the fungal isolate *T. albobiverticillius* HNB9 on plant growth and development (Table 5). Notably, the treated okra plants exhibited remarkable improvements, including a 16.9% increase in shoot length and a 47% increase in root length compared to the non-treated control plants. Additionally, the treated plants showed enhancements in other morphological traits, with a 31.5% increase in the number of leaves and a 48.6% improvement in leaf size (Table 5). Microscopic observations further confirmed the establishment of HNB9 spores in the cortical region of the host roots, indicating successful root colonization (Fig. 12). These findings highlight the efficacy of *T*. *albobiverticillius* HNB9 in promoting plant growth and development, particularly in okra plants.

**Table 5:** Effect of *T. albobiverticillius* HNB9 interaction on shoot length, root length and number of leaves of 15 day old Mustard plant in 1/2 × MS medium (*in-vitro*) and green house pot trails of Mustard (15 day old), maize (45 days old) and okra plants (45 day old).

	*In-vitro* mustard (15d)		Pot trials mustard (15d)		Pot trials maize (45d)		Pot trials okra (45d)	
	C	T	C	T	C	T	C	T
Shoot length (cm)	3.1 ± 0.34a	6.8 ± 0.3b	3.5 ± 0.35c	6.2 ± 0.37d	61.2 ± 4.5e	73.3 ± 6.3f	48.7 ± 1.4g	57.2 ± 2.7h
Root length (cm)	2.4 ± 0.8a	4.6 ± 0.7b	2.6 ± 0.7c	4.2 ± 0.3d	41.8 ± 3.01e	55.3 ± 3.7f	69.9 ± 2.4g	95.2 ± 3.1h
Number of leaves	2.7 ± 0.5a	5 ± 1.0b	3.3 ± 0.5c	5.7 ± 0.5d	5 ± 1.0e	7 ± 1.0f	8 ± 1.0g	11.7 ± 0.6h

Data in the study was collected in triplicates and represented as average ± SD. Different letters within a parameter indicate the values were significant at *P* < *0.05* as determined by Analysis of Variance (ANOVA).

## Discussion

In Stoll's study of rice and its microbial pathogens, a distinctive fungus with a tendency for producing pink exudates caught attention^36^. This fungus was later Identified as *Penicillium purpureogenum* by Stoll, its characterization was based on morphological traits, ultrastructure, and the unique ability to generate dark pink extracellular metabolites. The taxonomic shift occurred with the introduction of the genus *Talaromyces* to distinguish between *Aspergillus* and *Penicillium* species, leading to the renaming of *P*. *purpureogenum* as *T*.*purpureogenus*^37^.

The production of red pigments by *Talaromyces* species, including HNB9, *T. albobiverticillius*, and *T. rubrifaciens*, initially caused confusion and misidentification. However, a detailed comparison revealed that HNB9 shares more morphological similarities with *T. albobiverticillius* than *T. rubrifaciens* (Tables 6, 7). General morphological features, such as the presence of ascomata, strictly biverticillate conidiophores, and smooth-walled stipes, were more closely aligned between HNB9 and *T. albobiverticillius*. Despite slightly larger metulae in HNB9, the overall morphology resembled that of *T. albobiverticillius*, whereas *T. rubrifaciens* displayed distinctive characteristics^38,39^.

**Table 6:** Comparison of Isolate_HNB9 with *T. albobiverticillius* and *T. rubrifaciens* on different growth media (25 °C, 7 days).

Growth media	Isolate_HNB9	*T. albobiverticillius*	*T. rubrifaciens*
PDA	Colonies are 24–30 mm in diameter, White mycelia, floccose mycelia present at centre, exudate present and soluble pigment present, luscious growth, conidia green in colour and in abundance. Surface colour is white to red, reverse colour is reddish brown with radially furrow zonation and dense sporulation	Colonies are 21–24 mm in diameter. Surface colour is white to green, reverse colour is red with dense sporulation	NA
MEA	Colonies are 22–26 mm in diameter. Colonies are pinkish in colour because of exudates diffusing into mycelia. Mycelia white, texture velvety overlaying floccose, conidia green, and exudate red droplets. Surface colour is white to red, reverse colour is reddish brown, concentric rings present with dense sporulation	Colonies are 24–28 mm in diameter. Surface colour is white to red, reverse colour is red, radially furrowed zonation with dense sporulation. Colonies are pinkish colour because of exudates diffusing into mycelia. Mycelia white, texture velvety overlaying floccose, conidia bluish green and exudate red droplets	Colonies are 15–18 mm in diameter. Surface colour is white to green, reverse colour is brownish red with dense sporulation. Conidiogenesis abundant, conidia are greyish green to bluish green in colour, abundant red exudate, and abundant red soluble pigment
CYA	Colonies are 22–26 mm in diameter. Mycelia white, moderate sporulation, conidia yellow in colour, exudate present and soluble pigment present, luscious growth. Surface colour is white to red, reverse colour is red with radially furrow zonation	Colonies are 18–20 mm in diameter. Mycelia white, conidia dark to dull green in colour, exudate absent and soluble pigment red is present. Surface colour is white to red, reverse colour is red with heavy wrinkle zonation and dense sporulation	Colonies are 12–14 mm in diameter, restricted growth, mycelia yellow to white, conidia greenish grey to dull green in colour, no exudate, no soluble pigment. Surface colour is yellow to green, reverse colour is reddish brown with dense sporulation
DG18	Colonies are 8–12 mm in diameter. White mycelia, floccose mycelia present at centre, exudate absent, soluble pigment present, and restricted growth. Surface colour is white to red, reverse colour is white to brown with radial zonation and poor sporulation	Colonies are 24–36 mm in diameter. Mycelia white, floccose mycelia present at centre, conidia are greyish green, exudates absent and soluble pigment absent. Surface colour is green to red, reverse colour is red with radially furrow zonation and dense sporulation	Colonies are 4–5 mm in diameter, restricted growth, conidiogenesis sparse or not present on entire surface, no exudate, no soluble pigment. Surface colour is white to green, reverse colour is white with low sporulation
CYAS	no growth	no growth	NA
OMA	Colonies are 15–18 mm in diameter, White mycelia, low sporulation, conidia green, exudate absent, soluble pigment present, restricted growth of mycelia. Surface colour is white to green, reverse colour is white to brown with radially furrow zonation	Colonies are 23–28 mm in diameter, white mycelia, sporulation dense, conidia are blackish green, exudates absent and soluble pigment absent. Surface colour is white to green, reverse colour is white to brown with radially furrow zonation	NA
CREA	Colonies are < 1 mm in diameter. Surface and reverse colour is white, poor growth and no sporulation	NA	No growth

N/A designates data not available.

**Table 7:** Comparative table of general morphological features of the isolate HNB9 with respect to *T. albobiverticillius and T. rubrifaciens*.

General morphological features	Isolate_HNB9	*T. albobiverticillius*	*T. rubrifaciens*
Ascomata	Present (40.38 μm)	Present	Absent
Ascospores	Present (2.05 μm)	N/A	N/A
Conidiophores	Strictly biverticillate and broad penicillia, arising from surface hyphae with long stipes and smooth-walled	Strictly biverticillate, subterminal branches absent	Strictly biverticillate
Stipes	Smooth-walled, 158.8–160 μm long and 4.18–4.2 μm wide	Smooth-walled, 200–380 × 2.5–3.5 µm	Smooth-walled to finely roughened, 115–230 × 2.5–3.5 μm
Metulae	9–15 per stipe, 9.09 μm long	In verticals, 3–6, 8–12 × 1.5–4.5 μm	Hyaline, 9–15 per stipe, 4–5(–6) × 2.5–3.5 μm
Phialides	6–7 per metula, 8.8 μm long	Acerose, 3–7 per metula, 8–13.5 × 2–3 μm	Usually cylindrical, with or without short collula, 6–10 per metulae, phialides 3–5 × 2–3 μm
Conidia	Globose to ellipsoidal, smooth-walled, 2.41 μm wide	Smooth to finely roughened, spheroid to subglobose, in some isolates fusiform, 2–3.5 (4) × 1.5–2.5 μm	Globose to ellipsoidal, 2–4 × 2–3 μm, smooth-walled

N/A designates data not available.

Further comparisons of colony morphology on different media (Tables 6, 7) reaffirmed the closer relationship between HNB9 and *T. albobiverticillius*. Similar growth rates, colony sizes, mycelial texture, color, and sporulation were observed in both isolates, whereas *T. rubrifaciens* exhibited differences in these aspects^39^. This morphological evidence strongly suggests that HNB9 is closely associated with *T. albobiverticillius*.

Notably, HNB9 exhibits plant growth-promoting properties absent in *T. albobiverticillius* or *T*. *rubrifaciens*. Confocal images stained with a fungal-specific dye (WGA Alexa Fluor 488) revealed germ tube formation from spores, indicating enhanced penetration into plant roots. This aligns with the characteristics of other plant growth-promoting fungal root endophytes like *S. indica*^13–16^. Furthermore, a literature review emphasized the morphological similarities between *T*. *albobiverticillius* and *T. rubrifaciens*, supporting the findings of the present study^38,39^.

Pairwise identity values and phylogenetic analysis suggested a close relationship and shared ancestry among *T. rubrifaciens*, *T. albobiverticillius*, *T. heiheensis*, and isolate HNB9. This genetic relatedness is reflected in the distinct branches or clades formed in the phylogenetic analysis, leading to the synonymization of *T. rubrifaciens* with *T. albobiverticillius*^38^.

Contrary to earlier reviews that did not highlight plant growth-promoting activity in these species, the current study demonstrated that *T. albobiverticillius* HNB9 colonizes plant roots, enhancing overall plant health and growth. Confocal images provided insights into its penetration into plant roots, resembling the behavior of other plant growth-promoting fungal root endophytes^13–16^. Similar observations were made for other *Talaromyces* species, highlighting their potential for promoting plant growth and tolerance to various stresses^40–48^. Furthermore, *T. albobiverticillius* HNB9 exhibited additional plant growth-promoting properties, including IAA production, zinc, phosphorus, and silica solubilization.

The investigation into the red pigment produced by *T. albobiverticillius* revealed a complex mixture of Azaphilones, primarily found in Monascus species. Azaphilones are known for their diverse physiological activities, including anti-inflammatory, anticancer, and antimicrobial properties. The compounds can also react with amino acids, nucleic acids, and proteins, promoting the production of vinylogous γ-pyridones^49–55^. *T. albobiverticillius* and related species are known for producing orange, yellow, and red pigments belonging to two groups of azaphilone polyketides: mitorubrins and Monascus red pigments^56,57^.

Many fungal strains naturally produce azaphilones, contributing to the distinct coloration of fungal secondary metabolites. These compounds, absent in plants, can be synthesized in significant quantities through liquid fermentation of *Talaromyces* or *Penicillium* fungal strains^58^. For instance, *T. atroroseus*, identified in 2013, produces red diffusible pigments containing azaphilones, mitorubrins, and Monascus pigments, excluding most mycotoxins produced by other *Talaromyces* species^59^.

Additionally, *T. purpureogenus* demonstrated the synthesis of biogenic silver nanoparticles (Tp-AgNPs) from its mycelial extract, displaying anti-proliferating, wound healing, and antibacterial properties^60^. Endophytic strains like *T. assiutensis*, CPEF04, isolated from the roots of the mangrove plant *Avicennia marina*, exhibited anticancer and antimicrobial properties^61^. Similarly, *T. flavus*, isolated from the healthy leaves of the mangrove *Sonneratia apetala*, produced potent antitumor natural products^62^.

Moreover, *T. wortmanii*, isolated from an endophytic strain, displayed strong antimicrobial and anti-inflammatory activities^63,64^. *T*. *radicus*–Crp20, isolated from *Catharanthus roseus*, produced significant amounts of anticancer compounds vincristine and vinblastine in liquid cultures^65^. Various *Talaromyces* strains isolated from different plants exhibited polyketides with antifungal activity against plants and human pathogens^66^.

Phenolic compounds isolated from the *Punica granatum* fruit endophyte *T. purpureogenus* displayed significant activity against methicillin-resistant *Staphylococcus aureus* strain ATCC 700699^67^. Plant root endophytes, including *Talaromyces* species, contribute to plant growth, yield enhancement, and tolerance to biotic and abiotic stresses. They also produce diverse low molecular weight secondary metabolites, both in nature and in pure cultures, with potential pharmaceutical applications^68–71^.

Unlike conventional drug discovery methods, which often produce random products, plant root endophytes synthesize secondary metabolites through long-term adaptation processes for specific functions in their biotopes^72^. A new peptide termed cryptocandin, isolated from the fungal root endophyte *Cryptosporiopsis quercina*, exhibited excellent antimycotic activity against human pathogenic *Candida albicans* and *Trichophyton* spp.^73^. Another group of antifungal compounds known as pseudomycin, isolated from plant-associated pseudomonad, was found effective against various fungi^74–76^.

Fungal endophytes are also studied for their production of anticancerous compounds such as Paclitaxel, isolated from the endophytic fungus *T. andreanae*, used to treat tissue proliferating diseases in humans^77^. Secondary metabolites from endophytic fungi tend to act against agriculturally important pests and insects. Nodulisporic acids, novel indole diterpenes isolated from the fungal endophyte *Nodulisporium sp*., exhibit insecticidal properties^78,79^.

The present study underscores the significance of plant root colonizing endophytes, like *T. albobiverticillius* HNB9, as abundant sources of genetically diverse and novel natural compounds. These compounds hold promise for addressing various human ailments and discovering treatments for currently incurable diseases. Ongoing research is focused on exploring the agro-economic aspects and the ability of *T. albobiverticillius* HNB9 to colonize roots, enhance crop growth, and induce biotic and abiotic stress tolerance in host plants through the secretion of various plant growth-promoting compounds.

### Supplementary Information

Supplementary Tables.

## Data Availability

(1) The datasets generated and/or analysed during the current study are available in the National Center for Biotechnology Information (NCBI) repository and the corresponding accession numbers for the sequences are as follows: Sequence: HNB9_*BenA*_contig, HNB9_*CaM*_contig, HNB9_*RPB2*_contig, HNB9_ITS_contig have Accession number: ON406962, ON406963, ON406964, ON261679. (2) Type culture associated with the study has been submitted to the National Agriculturally Important Microbial Culture Collection (NAIMCC), which is part of the ICAR-National Bureau of Agriculturally Important Microorganisms (NBAIM) *Kushmaur*, *Mau Nath Bhanjan* Uttar Pradesh, India. The accession number assigned to the type culture is NAIMCC-SF-0025.
