# Supplementary material for: Isolation and characterization of a newly discovered plant growth-promoting endophytic fungal strain from the genus Talaromyces
Source: Sci Rep. 2024 Mar 12;14:6022. doi: 10.1038/s41598-024-54687-5 (PMC10933278; doi:10.1038/s41598-024-54687-5)
Supplement: Supplementary file 1 — Supplementary Tables. [file 41598_2024_54687_MOESM1_ESM.docx]

**SUPPLEMENTARY DATA**

**Supplementary Table 1:** Primers used in this study for amplification and sequencing.

| **Locus** | **Primer** | **Sequence (5’-3’)** | **Annealing Temperature** | **Fragment Size (bp)** |
| --- | --- | --- | --- | --- |
| *RPB2* | 5F_Eur | (Fwd) GAYGAYCGKGAYCAYTTCGG | 48-51 | Ca. 1220 |
|  | 7CR_Eur | (Rev) CCCATRGCYTGYTTRCCCAT |  |  |
| *BenA* | T10 | (Fwd) ACGATAGGTTCACCTCCAGAC | 55 | Ca. 570–620 |
|  | Bt2b | (Rev) ACCCTCAGTGTAGTGACCCTTGGC |  |  |
| *ITS* | V9G | (Fwd) TTACGTCCCTGCCCTTTGTA | 55 | Ca. 1000 |
|  | LS266 | (Rev) GCATTCCCAAACAACTCGACTC |  |  |
| *Cmd* | Cmd5 | (Fwd) CCGAGTACAAGGARGCCTTC | 55 | Ca. 630 |
|  |  | (Rev) CCGATRGAGGTCATRACGTGG |  |  |

**Supplementary Table 2.** Sequence data sets and models used in phylogeny

| **Locus name** | **No. of Seq.** | **No. of alignment position used in the final data set** | **Model for BI** |
| --- | --- | --- | --- |
| *ITS* | 36 | 913 | K2 + G |
| *BenA* | 38 | 573 | K2 + G + I |
| *CaM* | 39 | 620 | K2 + G + I |
| *RPB2* | 34 | 1004 | K2 + G + I |
| Full names of the used models: K2+I (Kimura 2-parameter with Invariant sites); K2+G+I (Kimura 2-parameter with Invariant sites and Gamma distribution) | | | |
|  |  |  |  |

| **Supplementary Table 3.** Fungal species identified on the basis of HNB9_*ITS* sequence BLAST against GenBank database | | | | | | | |
| --- | --- | --- | --- | --- | --- | --- | --- |
| **S.No.** | **Fungal  species** | **Phylum** | **Strains /  Isolates** | **GenBank accession no.^a^** | **Identity ^b^ (%)** | **Accession  sequence size** | **Section** |
| 1 | *T. rubrifaciens* | Ascomycota | CGMCC 3.17658 | KR855658.1 | 100 | 501 | sect. *Trachyspermi* |
| 2 | *T. albobiverticillius* | Ascomycota | 900890701 | HQ605705.1 | 98.9 | 545 | sect. *Trachyspermi* |
| 3 | *T. heiheensis* | Ascomycota | HMAS 248789 | KX447526.1 | 98.19 | 550 | sect. *Trachyspermi* |
| 4 | *T. solicola* | Ascomycota | Pen193 | FJ160264.1 | 97.5 | 518 | sect. *Trachyspermi* |
| 5 | *T. catalonicus* | Ascomycota | FMR 16441 | LT899793.1 | 97.44 | 433 | sect. *Trachyspermi* |
| 6 | *T. erythromellis* | Ascomycota | CBS 644.80 | JN899383.1 | 96.22 | 703 | sect. *Trachyspermi* |
| 7 | *T. albisclerotius* | Ascomycota | CBS 141839 | MN864276.1 | 93.85 | 580 | sect. *Trachyspermi* |
| 8 | *T. clemensii* | Ascomycota | PPRI 26753 | MK951940.1 | 93.39 | 739 | sect. *Trachyspermi* |
| 9 | *T. muroii* | Ascomycota | CBS 756.96 | MN431394.1 | 92.85 | 883 | sect. *Talaromyces* |
| 10 | *T. amyrossmaniae* | Ascomycota | NFCCI 1919 | MH909062.1 | 92.77 | 552 | sect. *Trachyspermi* |
| 11 | *T. calidicanius* | Ascomycota | CBS 112002 | JN899319.1 | 92.75 | 736 | sect. *Talaromyces* |
| 12 | *T. striatoconidius* | Ascomycota | CBS 550.89 | MN431418.1 | 92.63 | 1335 | sect. *Talaromyces* |
| 13 | *T. atroroseus* | Ascomycota | DTO 390-I4 | MN788119.1 | 92.52 | 894 | sect. *Trachyspermi* |
| 14 | *T. diversus* | Ascomycota | CBS 320.48 | KJ865740.1 | 92.52 | 760 | sect. *Trachyspermi* |
| 15 | *T. viridulus* | Ascomycota | CBS 252.87 | JN899314.1 | 92.41 | 740 | sect. *Talaromyces* |
| 16 | *T. duclauxii* | Ascomycota | CBS 322.48 | JN899342.1 | 92.12 | 734 | sect. *Talaromyces* |
| 17 | *T. minnesotensis* | Ascomycota | DI16-144 | LT558966.1 | 92.08 | 1088 | sect. *Trachyspermi* |
| 18 | *T. minioluteus* | Ascomycota | CBS 642.68 | JN899346.1 | 92.02 | 760 | sect. *Trachyspermi* |
| 19 | *T. udagawae* | Ascomycota | CBS 579.72 | JN899350.1 | 91.85 | 754 | sect. *Trachyspermi* |
| 20 | *T. liani* | Ascomycota | CBS 225.66 | JN899395.1 | 91.74 | 736 | sect. *Talaromyces* |
| 21 | *T. alveolaris* | Ascomycota | DI16-147 | LT558969.1 | 91.69 | 1080 | sect. *Talaromyces* |
| 22 | *T. convolutus* | Ascomycota | CBS 100537 | NR_137157.1 | 91.32 | 480 | sect. *Trachyspermi* |
| 23 | *T. chongqingensis* | Ascomycota | CS26-67 | MZ358001.1 | 91.26 | 960 | sect. *Trachyspermi* |
| 24 | *T. purpureogenus* | Ascomycota | CBS 286.36 | JN899372.1 | 91.25 | 737 | sect. *Talaromyces* |
| 25 | *T. guatemalensis* | Ascomycota | CCF 6215 | MN322789.1 | 91.11 | 549 | sect. *Trachyspermi* |
| 26 | *T. stipitatus* | Ascomycota | CBS 375.48 | JN899348.1 | 90.84 | 734 | sect. *Talaromyces* |
| 27 | *T. mycothecae* | Ascomycota | URM 7622 | MF278326.1 | 90.76 | 437 | sect. *Talaromyces* |
| 28 | *T. brasiliensis* | Ascomycota | URM 7618 | MF278323.1 | 90.72 | 438 | sect. *Trachyspermi* |
| 29 | *T. verruculosus* | Ascomycota | NRRL1050 | KF741994.1 | 89.86 | 550 | sect. *Talaromyces* |
| 30 | *T. austrocalifornicus* | Ascomycota | S3D | MW897776.1 | 89.68 | 620 | sect. *Trachyspermi* |
| 31 | *T. speluncarum* | Ascomycota | FMR 16671 | LT985890.1 | 89.54 | 482 | sect. *Trachyspermi* |
| 32 | *T. systylus* | Ascomycota | Unknown | KP026917.1 | 88.98 | 579 | sect. *Trachyspermi* |
| 33 | *T. aerius* | Ascomycota | CBS 140611 | KU866647.1 | 88.83 | 581 | sect. *Trachyspermi* |
| 34 | *T. assiutensis* | Ascomycota | CBS 147.78 | JN899323.1 | 87.08 | 502 | sect. *Trachyspermi* |
| 35 | *T. subericola* | Ascomycota | FMR 15656 | LT985888.1 | 86.61 | 467 | sect. *Trachyspermi* |
| **^a^** GenBank accession no. based on *ITS* sequences available in the GenBank database | | | | | | |  |
| **^b^** % Identity of *ITS* sequences available in GenBank, compared to HNB9_*ITS* sequence | | | | | | |  |

| **Supplementary Table 4.** Fungal species identified on the basis of HNB9_*BenA* sequence BLAST against GenBank database | | | | | | | |
| --- | --- | --- | --- | --- | --- | --- | --- |
| **S.No.** | **Fungal  species** | **Phylum** | **Strains /  Isolates** | **GenBank accession no.^a^** | **Identity ^b^ (%)** | **Accession  sequence size** | **Section** |
| 1 | *T. albobiverticillius* | Ascomycota | DTO 270B8 | KJ775225.1 | 99.77 | 437 | sect. *Trachyspermi* |
| 2 | *T. rubrifaciens* | Ascomycota | CGMCC 3.17658 | KR855648.1 | 98.99 | 397 | sect. *Trachyspermi* |
| 3 | *T. erythromellis* | Ascomycota | CBS 644.80 | HQ156945.1 | 96.4 | 444 | sect. *Trachyspermi* |
| 4 | *T. solicola* | Ascomycota | CV191 | GU385731.1 | 95.99 | 398 | sect. *Trachyspermi* |
| 5 | *T. catalonicus* | Ascomycota | FMR 16441 | LT898318.1 | 95.83 | 587 | sect. *Trachyspermi* |
| 6 | *T. heiheensis* | Ascomycota | HMAS 248789 | KX447525.1 | 95.54 | 447 | sect. *Trachyspermi* |
| 7 | *T. aerius* | Ascomycota | DTO 317-C7 | KU866835.1 | 94.21 | 430 | sect. *Trachyspermi* |
| 8 | *T. solicola* | Ascomycota | Unknown | LR535945.1 | 92.68 | 321 | sect. *Trachyspermi* |
| 9 | *T. convolutus* | Ascomycota | CBS 100537 | KF114773.1 | 89.97 | 328 | sect. *Trachyspermi* |
| 10 | *T. austrocalifornicus* | Ascomycota | CBS 644.95 | KJ865732.1 | 89.96 | 456 | sect. *Trachyspermi* |
| 11 | *T. diversus* | Ascomycota | CBS 320.48 | KJ865723.1 | 87.5 | 457 | sect. *Trachyspermi* |
| 12 | *T. atroroseus* | Ascomycota | CBS133442 | KF114789.1 | 87.5 | 341 | sect. *Trachyspermi* |
| 13 | *T. clemensii* | Ascomycota | PPRI 26753 | MK951833.1 | 87.26 | 422 | sect. *Trachyspermi* |
| 14 | *T. amyrossmaniae* | Ascomycota | NFCCI 1919 | MH909064.1 | 86.39 | 334 | sect. *Trachyspermi* |
| 15 | *T. guatemalensis* | Ascomycota | CCF 6215 | MN329687.1 | 86.36 | 412 | sect. *Trachyspermi* |
| 16 | *T. chongqingensis* | Ascomycota | CS26-75 | MZ361346.1 | 86.34 | 438 | sect. *Trachyspermi* |
| 17 | *T. chongqingensis* | Ascomycota | CS26-73 | MZ361345.1 | 86.34 | 438 | sect. *Trachyspermi* |
| 18 | *T. chongqingensis* | Ascomycota | CS26-63 | MZ361344.1 | 86.34 | 438 | sect. *Trachyspermi* |
| 19 | *T. chongqingensis* | Ascomycota | CS26-67 | MZ361343.1 | 86.34 | 438 | sect. *Trachyspermi* |
| 20 | *T. albisclerotius* | Ascomycota | CBS 141839 | MN863345.1 | 86.3 | 433 | sect. *Trachyspermi* |
| 21 | *T. minnesotensis* | Ascomycota | DI16-144 | LT559083.1 | 86.09 | 555 | sect. *Trachyspermi* |
| 22 | *T. minioluteus* | Ascomycota | CBS 642.68 | MN969409.1 | 86.06 | 436 | sect. *Trachyspermi* |
| 23 | *T. purpureogenus* | Ascomycota | KAS3773 | JF910281.1 | 84.99 | 473 | sect. *Talaromyces* |
| 24 | *T. assiutensis* | Ascomycota | CBS 147.78 | KJ865720.1 | 83.98 | 452 | sect. *Trachyspermi* |
| 25 | *T. udagawae* | Ascomycota | CBS 579.72 | KF114796.1 | 83.57 | 334 | sect. *Trachyspermi* |
| 26 | *T. brasiliensis* | Ascomycota | URM 7618 | LT855560.1 | 83.19 | 346 | sect. *Trachyspermi* |
| 27 | *T. systylus* | Ascomycota | Unknown | KR233838.1 | 82.8 | 471 | sect. *Trachyspermi* |
| 28 | *T. alveolaris* | Ascomycota | DI16-147 | LT559086.1 | 82.09 | 446 | sect. *Talaromyces* |
| 29 | *T. stipitatus* | Ascomycota | CBS 375.48 | KM111288.1 | 81.71 | 331 | sect. *Talaromyces* |
| 30 | *T. duclauxii* | Ascomycota | CBS 322.48 | JX091384.1 | 81.13 | 413 | sect. *Talaromyces* |
| 31 | *T. calidicanius* | Ascomycota | CBS 112002 | HQ156944.1 | 81.07 | 458 | sect. *Talaromyces* |
| 32 | *T. mycothecae* | Ascomycota | URM 7622 | LT855561.1 | 81.01 | 424 | sect. *Talaromyces* |
| 33 | *T. muroii* | Ascomycota | CBS 756.96 | KJ865727.1 | 81 | 435 | sect. *Talaromyces* |
| 34 | *T. striatoconidius* | Ascomycota | CBS 550.89 | MN969441.1 | 80.65 | 436 | sect. *Talaromyces* |
| 35 | *T. verruculosus* | Ascomycota | NRRL1050 | KF741928.1 | 80.31 | 421 | sect. *Talaromyces* |
| 36 | *T. liani* | Ascomycota | CBS 225.66 | JX091380.1 | 79.95 | 401 | sect. *Talaromyces* |
| 37 | *T. viridulus* | Ascomycota | CBS 252.87 | JX091385.1 | 79.78 | 398 | sect. *Talaromyces* |
| **^a^** GenBank accession no. based on *BenA* sequences available in the GenBank database | | | | | | |  |
| **^b^** % Identity of *BenA* sequences available in GenBank, compared to HNB9_*BenA* sequence | | | | | | |  |

| **Supplementary Table 5.** Fungal species identified on the basis of HNB9_*CaM* sequence BLAST against GenBank database | | | | | | | | | |
| --- | --- | --- | --- | --- | --- | --- | --- | --- | --- |
| **S.No.** | **Fungal  species** | **Phylum** | **Strains /  Isolates** | **GenBank accession no.^a^** | **Identity ^b^ (%)** | **Accession  sequence size** | **Section** | |  |
| 1 | *T. rubrifaciens* | Ascomycota | CGMCC 3.17658 | KJ885279.1 | 97.73 | 441 | sect. *Trachyspermi* | |  |
| 2 | *T. heiheensis* | Ascomycota | HMAS 248789 | KF741934.1 | 93.57 | 496 | sect. *Talaromyces* | |  |
| 3 | *T. albobiverticillius* | Ascomycota | CBS 133440 | KJ885260.1 | 93.44 | 557 | sect. *Trachyspermi* | |  |
| 4 | *T. amyrossmaniae* | Ascomycota | NFCCI 1919 | KJ885273.1 | 93.37 | 547 | sect. *Trachyspermi* | |  |
| 5 | *T. erythromellis* | Ascomycota | CBS 644.80 | KX961260.1 | 91.6 | 494 | sect. *Trachyspermi* | |  |
| 6 | *T. solicola* | Ascomycota | Unknown | KJ885270.1 | 91.44 | 400 | sect. *Trachyspermi* | |  |
| 7 | *T. clemensii* | Ascomycota | PPRI 26753 | KJ885268.1 | 90.77 | 425 | sect. *Trachyspermi* | |  |
| 8 | *T. solicola* | Ascomycota | DAOM 241015 | KR855653.1 | 90.55 | 556 | sect. *Trachyspermi* | |  |
| 9 | *T. catalonicus* | Ascomycota | FMR 16441 | KU866731.1 | 89.31 | 488 | sect. *Trachyspermi* | |  |
| 10 | *T. aerius* | Ascomycota | DTO 317-C7 | KX447532.1 | 89.19 | 460 | sect. *Trachyspermi* | |  |
| 11 | *T. albisclerotius* | Ascomycota | CBS 141839 | LT795604.1 | 86.83 | 605 | sect. *Trachyspermi* | |  |
| 12 | *T. subericola* | Ascomycota | FMR 15656 | LT899775.1 | 86.05 | 444 | sect. *Trachyspermi* | |  |
| 13 | *T. austrocalifornicus* | Ascomycota | CBS 644.95 | MH909068.1 | 85.25 | 465 | sect. *Trachyspermi* | |  |
| 14 | *T. viridulus* | Ascomycota | CBS 252.87 | MK951906.1 | 84.62 | 461 | sect. *Trachyspermi* | |  |
| 15 | *T. convolutus* | Ascomycota | CBS 100537 | MN329688.1 | 84.43 | 464 | sect. *Trachyspermi* | |  |
| 16 | *T. brasiliensis* | Ascomycota | URM 7618 | MN863322.1 | 84.38 | 490 | sect. *Trachyspermi* | |  |
| 17 | *T. alveolaris* | Ascomycota | DI16-147 | MZ361350.1 | 84.36 | 524 | sect. *Trachyspermi* | |  |
| 18 | *T. guatemalensis* | Ascomycota | CCF 6215 | KF741947.1 | 84.31 | 660 | sect. *Talaromyces* | |  |
| 19 | *T. verruculosus* | Ascomycota | CBS 254.56 | KJ885258.1 | 83.73 | 480 | sect. *Trachyspermi* | |  |
| 20 | *T. muroii* | Ascomycota | CBS 756.96 | MN969316.1 | 83.48 | 481 | sect. *Trachyspermi* | |  |
| 21 | *T. calidicanius* | Ascomycota | CBS 112002 | KJ885261.1 | 83.18 | 457 | sect. *Trachyspermi* | |  |
| 22 | *T. systylus* | Ascomycota | Unknown | KF741943.1 | 83.14 | 489 | sect. *Talaromyces* | |  |
| 23 | *T. mycothecae* | Ascomycota | URM 7622 | KJ885257.1 | 83.06 | 463 | sect. *Talaromyces* | |  |
| 24 | *T. striatoconidius* | Ascomycota | CBS 550.89 | LT795596.1 | 82.99 | 484 | sect. *Talaromyces* | |  |
| 25 | *T. purpureogenus* | Ascomycota | CBS 286.36 | KJ885274.1 | 82.93 | 466 | sect. *Talaromyces* | |  |
| 26 | *T. diversus* | Ascomycota | CBS 320.48 | MN969360.1 | 82.93 | 471 | sect. *Talaromyces* | |  |
| 27 | *T. stipitatus* | Ascomycota | CBS 375.48 | LT985904.1 | 82.88 | 444 | sect. *Trachyspermi* | |  |
| 28 | *T. liani* | Ascomycota | CBS 225.66 | LR535946.1 | 82.79 | 484 | sect. *Trachyspermi* | |  |
| 29 | *T. assiutensis* | Ascomycota | CBS 147.78 | MZ361351.1 | 82.73 | 573 | sect. *Trachyspermi* | |  |
| 30 | *T. speluncarum* | Ascomycota | FMR 16671 | MZ361352.1 | 81.63 | 514 | sect. *Trachyspermi* | |  |
| 31 | *T. atroroseus* | Ascomycota | CBS133442 | MZ361353.1 | 81.42 | 487 | sect. *Trachyspermi* | |  |
| 32 | *T. minnesotensis* | Ascomycota | DI16-144 | LT855563.1 | 79.55 | 512 | sect. *Trachyspermi* | |  |
| 33 | *T. udagawae* | Ascomycota | CBS 579.72 | KR233837.1 | 79.48 | 487 | sect. *Trachyspermi* | |  |
| 34 | *T. minioluteus* | Ascomycota | CBS 642.68 | LT985906.1 | 79.41 | 491 | sect. *Trachyspermi* | |  |
| 35 | *T. chongqingensis* | Ascomycota | CS26-75 | KJ775418.1 | 79.14 | 497 | sect. *Trachyspermi* | |  |
| 36 | *T. chongqingensis* | Ascomycota | CS26-73 | LT855564.1 | 79.14 | 497 | sect. *Talaromyces* | |  |
| 37 | *T. chongqingensis* | Ascomycota | CS26-63 | KF741957.1 | 79.14 | 497 | sect. *Talaromyces* | |  |
| 38 | *T. chongqingensis* | Ascomycota | CS26-67 | KF741944.1 | 79.14 | 497 | sect. *Talaromyces* | |  |
| **^a^** GenBank accession no. based on *CaM* sequences available in the GenBank database | | | | | | | |  | |
| **^b^** % Identity of *CaM* sequences available in GenBank, compared to HNB9_*CaM* sequence | | | | | | | |  | |

| **Supplementary Table 6.** Fungal species identified on the basis of HNB9_*RPB2* sequence BLAST against GenBank database | | | | | | | |
| --- | --- | --- | --- | --- | --- | --- | --- |
| **S.No.** | **Fungal  species** | **Phylum** | **Strains /  Isolates** | **GenBank accession no.^a^** | **Identity ^b^ (%)** | **Accession  sequence size** | **Section** |
| 1 | *T. rubrifaciens* | Ascomycota | CGMCC 3.17658 | KR855663.1 | 99.11 | 821 | sect. *Trachyspermi* |
| 2 | *T. albobiverticillius* | Ascomycota | CBS 133440 | KM023310.1 | 98.47 | 852 | sect. *Trachyspermi* |
| 3 | *T. erythromellis* | Ascomycota | CBS 644.80 | KM023290.1 | 98 | 852 | sect. *Trachyspermi* |
| 4 | *T. heiheensis* | Ascomycota | HMAS 248789 | KX447529.1 | 97.31 | 1048 | sect. *Trachyspermi* |
| 5 | *T. catalonicus* | Ascomycota | FMR 16441 | LT899811.1 | 97.23 | 833 | sect. *Trachyspermi* |
| 6 | *T. solicola* | Ascomycota | DAOM 241015 | KM023295.1 | 95.95 | 843 | sect. *Trachyspermi* |
| 7 | *T. aerius* | Ascomycota | CBS 140611 | KU866991.1 | 95.51 | 895 | sect. *Trachyspermi* |
| 8 | *T. solicola* | Ascomycota | Unknown | LR535948.1 | 95.36 | 517 | sect. *Trachyspermi* |
| 9 | *T. amyrossmaniae* | Ascomycota | NFCCI 1919 | MH909066.1 | 91.53 | 893 | sect. *Trachyspermi* |
| 10 | *T. convolutus* | Ascomycota | CBS 100537 | JN121414.1 | 89.93 | 954 | sect. *Trachyspermi* |
| 11 | *T. udagawae* | Ascomycota | DTO 302-A8 | MN969148.1 | 89.77 | 948 | sect. *Trachyspermi* |
| 12 | *T. chongqingensis* | Ascomycota | CS26-75 | MZ361360.1 | 89.74 | 1081 | sect. *Trachyspermi* |
| 13 | *T. chongqingensis* | Ascomycota | CS26-73 | MZ361359.1 | 89.74 | 1081 | sect. *Trachyspermi* |
| 14 | *T. chongqingensis* | Ascomycota | CS26-63 | MZ361358.1 | 89.74 | 1081 | sect. *Trachyspermi* |
| 15 | *T. chongqingensis* | Ascomycota | CS26-67 | MZ361357.1 | 89.74 | 1081 | sect. *Trachyspermi* |
| 16 | *T. austrocalifornicus* | Ascomycota | CBS 644.95 | MN969147.1 | 89.66 | 949 | sect. *Trachyspermi* |
| 17 | *T. albisclerotius* | Ascomycota | CBS 141839 | MN863334.1 | 89.53 | 917 | sect. *Trachyspermi* |
| 18 | *T. minnesotensis* | Ascomycota | DI16-144 | LT795605.1 | 89.05 | 1161 | sect. *Trachyspermi* |
| 19 | *T. minioluteus* | Ascomycota | CBS 642.68 | JF417443.1 | 89 | 976 | sect. *Trachyspermi* |
| 20 | *T. diversus* | Ascomycota | CBS 320.48 | KM023285.1 | 88.95 | 852 | sect. *Trachyspermi* |
| 21 | *T. speluncarum* | Ascomycota | FMR 16671 | LT985911.1 | 88.82 | 852 | sect. *Trachyspermi* |
| 22 | *T. assiutensis* | Ascomycota | CBS 147.78 | KM023305.1 | 88.66 | 582 | sect. *Trachyspermi* |
| 23 | *T. clemensii* | Ascomycota | PPRI 26753 | MN418451.1 | 87.95 | 1081 | sect. *Trachyspermi* |
| 24 | *T. subericola* | Ascomycota | FMR 15656 | LT985909.1 | 87.85 | 852 | sect. *Trachyspermi* |
| 25 | *T. atroroseus* | Ascomycota | CBS133442 | KM023288.1 | 87.71 | 651 | sect. *Trachyspermi* |
| 26 | *T. guatemalensis* | Ascomycota | CCF 6215 | MN329689.1 | 87.59 | 1020 | sect. *Trachyspermi* |
| 27 | *T. brasiliensis* | Ascomycota | URM 7618 | MN969198.1 | 87.2 | 1003 | sect. *Trachyspermi* |
| 28 | *T. stipitatus* | Ascomycota | CBS 375.48 | KM023280.1 | 84.7 | 817 | sect. *Talaromyces* |
| 29 | *T. calidicanius* | Ascomycota | CBS 112002 | KM023311.1 | 84.61 | 763 | sect. *Talaromyces* |
| 30 | *T. purpureogenus* | Ascomycota | CBS 286.36 | JX315709.1 | 84.37 | 1038 | sect. *Talaromyces* |
| 31 | *T. duclauxii* | Ascomycota | CBS 322.48 | JN121491.1 | 83.86 | 954 | sect. *Talaromyces* |
| 32 | *T. mycothecae* | Ascomycota | URM 7622 | LT855567.1 | 83.43 | 706 | sect. *Talaromyces* |
| 33 | *T. verruculosus* | Ascomycota | AX2101 I | KJ476428.1 | 82.34 | 1034 | sect. *Talaromyces* |
| **^a^** GenBank accession no. based on *RPB2* sequences available in the GenBank database | | | | | | |  |
| **^b^** % Identity of *RPB2* sequences available in GenBank, compared to HNB9_*RPB2* sequence | | | | | | |  |

| **Supplementary Table 7:** Morphological characteristics of *T. albobiverticillius* HNB9 | | | | | | | | |
| --- | --- | --- | --- | --- | --- | --- | --- | --- |
| **Morphological features** | **Morphological parameters** | **Culture media (fungal culture has been grown for 7 days at a temperature of 25 °C)** | | | | | | |
|  |  | **PDA** | **MEA** | **CYA** | **DG18** | **CYAS** | **OMA** | **CREA** |
| Colony morphology | Size | 21–24 mm | 24–28 mm | 18–20 mm | 24–36 mm | No growth | 23–28 mm | N/A |
|  | Surface color | White to green | White to red | White to red | Green to red | N/A | White to green | N/A |
|  | Reverse color | Red | Red with radially furrowed zonation and dense sporulation | Red with heavy wrinkle zonation and dense sporulation | Red with radially furrowed zonation and dense sporulation | N/A | White to brown with radially furrow zonation | N/A |
| Hyphal characteristics | Sporulation | Dense | Dense | Dense | Dense | N/A | Dense | N/A |
|  | Exudate | N/A | Red droplets diffusing into mycelia | Absent | Absent | N/A | Absent | N/A |
|  | Pigment | N/A | N/A | Red is present | Absent | N/A | Absent | N/A |
|  | Mycelia Colour | N/A | White | White | White | N/A | White | N/A |
|  | Mycelia Texture | N/A | Velvety overlaying floccose | N/A | Floccose mycelia present at centre | N/A | N/A | N/A |
|  | Mycelia Growth | N/A | N/A | N/A | N/A | N/A | N/A | N/A |
| Conidial morphology | Color | N/A | Bluish green | Dark to dull green | Greyish green | N/A | Blackish green | N/A |
| ***N/A designates data not available*** | | | | | | | | |

| **Supplementary Table 8:** Morphological characteristics of *T*. *rubrifaciens* | | | | | | | | |
| --- | --- | --- | --- | --- | --- | --- | --- | --- |
| **Morphological features** | **Morphological parameters** | **Culture media (fungal culture has been grown for 7 days at a temperature of 25 °C)** | | | | | | |
|  |  | **PDA** | **MEA** | **CYA** | **DG18** | **CYAS** | **OMA** | **CREA** |
| Colony morphology | Size | N/A | 15-18 mm | 12-14 mm | 4-5 mm | N/A | N/A | No growth |
|  | Surface color | N/A | White to green | Yellow to green | White to green | N/A | N/A | N/A |
|  | Reverse color | N/A | Brownish red | Reddish brown | White | N/A | N/A | N/A |
| Hyphal characteristics | Sporulation | N/A | Dense | Dense | Low | N/A | N/A | N/A |
|  | Exudate | N/A | Abundant red | Absent | Absent | N/A | N/A | N/A |
|  | Pigment | N/A | Abundant red | Absent | Absent | N/A | N/A | N/A |
|  | Mycelia Colour | N/A | N/A | Yellow to white | N/A | N/A | N/A | N/A |
|  | Mycelia Texture | N/A | N/A | N/A | N/A | N/A | N/A | N/A |
|  | Mycelia Growth | N/A | N/A | N/A | N/A | N/A | N/A | N/A |
| Conidial morphology | Color | N/A | Greyish green to bluish green | Greenish grey to dull green | N/A | N/A | N/A | N/A |
| ***N/A designates data not available*** | | | | | | | | |
